# Pigeonpea leaves in orthopedic diseases: bioactive metabolites, pharmacological actions and therapeutic potential

**DOI:** 10.3389/fphar.2026.1918539

**Published:** 2026-10-07

**Authors:** Yuan Zeng, Weifeng Liang, Jiayi Chen, Feifan Luan, Zhuneng Guo, Chunwei Wei, Xusong Li, Chenxiao Zheng, Minhua Hu

**Affiliations:** 1 Zhongshan Hospital of Traditional Chinese Medicine Affiliated to Guangzhou University of Traditional Chinese Medicine, Zhongshan, China; 2 Zhongshan Hospital of Traditional Chinese Medicine, Zhongshan, China

**Keywords:** bone metabolism, osteoarthritis, osteonecrosis of the femoral head, osteoporosis, pigeonpea leaves, Tongluo Shenggu Capsule

## Abstract

Pigeonpea leaves are derived from *Cajanus cajan (L.)* Huth [Fabaceae] and have long been used in traditional medicine to relieve blood stasis, swelling, pain, and musculoskeletal disorders. Flavonoids, stilbenes, phenolic acids, and triterpenoids are among the main metabolite classes identified in pigeonpea leaves. Experimental studies have linked pigeonpea leaf-derived materials to changes in inflammatory and oxidative-stress-related outcomes, microcirculation, and bone metabolism. Accordingly, their potential relevance to orthopedic disorders, including osteonecrosis of the femoral head, osteoporosis, osteoarthritis, and bone defects, has attracted increasing interest. Reported preclinical effects include changes in osteogenic differentiation, osteoclast formation, local blood supply, lipid metabolism, and inflammation- or oxidative-stress-related tissue injury. Among the investigated materials, total-flavonoid fractions, stilbene-rich extracts, the purified metabolite cajanin, and Tongluo Shenggu Capsule have shown biological activity in experimental models. This review summarizes the current research on pigeonpea leaves and their extracts, fractions, purified metabolites, and preparations in orthopedic disorders and discusses their possible mechanisms of action, potential relevance to orthopedic disorders, limitations of the available evidence, and remaining research gaps.

## 1 Introduction

Pigeonpea (*Cajanus cajan (L.)* Huth [Fabaceae]) is native to the Indian subcontinent and is now widely cultivated across tropical and subtropical regions, including Southeast Asia, Africa, Latin America, and the Caribbean (Royal Botanic Gardens and Kew, 2026; Full et al., 2019). Genetic and archaeobotanical evidence supports its domestication in India and its long history as a pulse crop in South Asia (Full et al., 2019; Kassa et al., 2012). Its seeds are widely consumed in South and Southeast Asia, most commonly as split dhal; immature green seeds and other local preparations are also consumed in parts of Southeast Asia (Full et al., 2019). Other plant parts have distinct uses, with the leaves used as fodder and the woody stems as household fuel (Full et al., 2019). Although relevant to the history and regional importance of the species, these food and agricultural uses do not provide evidence for the orthopedic efficacy of pigeonpea leaves.

In China, pigeonpea leaves have been investigated as a medicinal plant resource, with early phytochemical studies reporting the isolation of flavonoid metabolites from the leaves (Lin et al., 1999). From the perspective of traditional Chinese medicine (TCM), the development and progression of orthopedic diseases are often associated with liver and kidney deficiency, insufficient nourishment of “Qi” and “Blood”, and blood stasis obstructing the collaterals; prolonged disease may further lead to inadequate nourishment of tendons and bones, thereby impairing bone and joint function (Cai et al., 2020). The traditional functions of pigeonpea leaves, including promoting blood circulation, unblocking meridians, reducing swelling and relieving pain, are therefore conceptually related to the pathological features of orthopedic conditions, such as blood stasis, local swelling and pain, and impaired tissue repair.

With advances in TCM pharmacology and natural product chemistry, increasing attention has been paid to the metabolite profile and pharmacological properties of pigeonpea leaves. Previous studies have identified several classes of metabolites, particularly flavonoids, stilbenes, and phenolic acids. Studies using pigeonpea leaf extracts, enriched fractions, and purified metabolites have reported anti-inflammatory, antioxidant, hemorheological, and bone-metabolic outcomes in experimental models (Cai et al., 2020; Wang et al., 2025; Monrroy and García, 2025; Hou et al., 2024; Sun et al., 2019). Because of these properties, pigeonpea leaf extracts and major reported metabolites have gradually attracted attention in orthopedic research. Related preparations, particularly Tongluo Shenggu Capsule, which contains pigeonpea leaf as its sole botanical drug, have been studied in several orthopedic conditions, including osteonecrosis of the femoral head (Yuan et al., 2004; Hai and Li, 2014), osteoporosis, osteoarthritis, and bone defect repair (Table 1). These studies provide emerging experimental evidence for the modern interpretation and potential translational application of this traditional medicinal resource.

**TABLE 1:** Pigeonpea leaf-derived materials in orthopedic diseases: evidence levels and proposed mechanisms.

Disease area	Investigated material	Experimental evidence	Principal effects	Main pathways or markers
Osteonecrosis of the femoral head	Total flavonoids from pigeonpea leaves; stilbene-rich extracts; cajanin; cajaninstilbene acid; tongluo shenggu capsule	Evidence level: Human, animal and cell (limited clinical reports; steroid-induced ONFH animal models; hyperlipidemic models; HUVECs; BMSCs; osteoclast models; transcriptomic and proteomic analyses)	Preclinical studies suggest improvements in dyslipidemia, blood viscosity and endothelial injury; promotion of angiogenesis and osteogenic differentiation; and inhibition of adipogenic differentiation and inflammatory signaling. Anti-apoptotic evidence is mainly indirect (Yuan et al., 2004; Hai and Li, 2014; Luo et al., 2009; Luo, 2008; Liu et al., 2008; Sun, 2018; Zhao et al., 2020; Wang et al., 2021; Liu et al., 2021; Chen et al., 2016; Yang et al., 2023; Fan et al., 2021; Jiang et al., 2014; Liu et al., 2014; Ma et al., 2013)	TC, TG, LDL-C; PT/APTT/TT; VEGF/vegfr2-MEK/ERK; ET-1/NO; RUNX2/OSX, ABCB1 and PPARγ; TLR4/MyD88/NF-κb; caspase-3/-9; HSP/COP9
Osteoporosis	Cajaninstilbene acid and other stilbene metabolites; cajanine; pigeonpea-leaf extracts; tongluo shenggu capsule	Evidence level: Cell and animal (RANKL-induced osteoclastogenesis; ovariectomy and stress-deprivation bone-loss models; hBMSCs and osteoblast/osteoclast-like cell models)	Promotes osteogenic differentiation and mineralization and suppresses osteoclast differentiation and bone resorption in preclinical models. Differences among ovariectomy, mechanical-unloading and cell models limit generalization, and the MTT proliferation result requires confirmation with an orthogonal assay (Sun et al., 2019; Zheng et al., 2007a; Liu et al., 2011; Ma, 2024; Zhao et al., 2019; Ma et al., 2026; Zheng et al., 2007b)	RUNX2/OSX; ALP, OCN and OPN; ER/PTH; RANKL/RANK-MAPK/NF-κb-ca^2+^/nfatc1; Siglec15/NFATc1
Osteoarthritis	Tongluo shenggu capsule	Evidence level: Cell and animal (MIA-induced rat OA; IL-1β-stimulated SW1353 cells; rabbit full-thickness cartilage-defect model; H_2_O_2_-induced oxidative injury in chondrocytes)	Reduces inflammatory and cartilage-catabolic markers and enhances antioxidant responses in preclinical models. The acute cartilage-defect and oxidative-injury models provide adjacent or mechanistic evidence but do not establish disease-modifying efficacy in chronic human OA (Li Y. et al., 2020; Tang et al., 2010; Lin et al., 2020)	IL-1β; wnt/β-catenin; MMP-13; Keap1/Nrf2/ARE; nrf2/HO-1; GCLC and NQO1
Bone-defect repair	Total flavonoids from pigeonpea leaves combined with an α-calcium sulfate hemihydrate scaffold	Evidence level: Animal (rat critical-size calvarial defect model using a composite scaffold)	Enhances new-bone formation compared with the scaffold alone. Evidence is limited to one preclinical composite-material study, which cannot fully separate the effect of total flavonoids from that of the scaffold (Li X. et al., 2020)	New-bone formation and defect filling; no specific molecular pathway was established in the cited study

Abbreviations: ABCB1, ATP-binding cassette subfamily B member 1; ALP, alkaline phosphatase; APTT, activated partial thromboplastin time; BMSC, bone marrow mesenchymal stem cell; ER, estrogen receptor; ET-1, endothelin-1; GCLC, glutamate-cysteine ligase catalytic subunit; H_2_O_2_, hydrogen peroxide; HSP, heat shock protein; HUVEC, human umbilical vein endothelial cell; LDL-C, low-density lipoprotein cholesterol; MIA, monosodium iodoacetate; MMP-13, matrix metalloproteinase-13; NFATc1, nuclear factor of activated T cells cytoplasmic 1; NO, nitric oxide; NQO1, NAD(P)H quinone dehydrogenase 1; OA, osteoarthritis; OCN, osteocalcin; ONFH, osteonecrosis of the femoral head; OPN, osteopontin; OSX, osterix; PT, prothrombin time; RANKL, receptor activator of nuclear factor-κB ligand; RUNX2, runt-related transcription factor 2; TC, total cholesterol; TG, triglyceride; TT, thrombin time; VEGF, vascular endothelial growth factor; VEGFR2, vascular endothelial growth factor receptor 2. Evidence types are identified as human, animal, cell or indirect/adjacent evidence; this indicates translational proximity rather than an automatic risk-of-bias judgement. Detailed study-level material, model, dose, controls, duration and limitations are provided in Supplementary Table S2.

Although current studies have provided preliminary evidence for the bone-protective effects of pigeonpea leaves in relation to osteogenic differentiation, osteoclast activity, angiogenesis, inflammatory responses, and oxidative stress, their pharmacodynamic basis, key molecular targets, and clinical evidence remain insufficiently defined. Therefore, this review summarizes current research on pigeonpea leaves and the associated extracts, fractions, purified metabolites, and preparations in orthopedic diseases, with the aim of informing the further development of pigeonpea leaf-derived medicinal resources while highlighting their potential relevance to orthopedic disorders and the limitations affecting clinical translation.

## 2 Literature search and study selection

### 2.1 Search strategy

This review was conducted as a critical narrative review informed by a structured literature search. It was not designed as a systematic review or meta-analysis because of the limited number and substantial heterogeneity of the available studies in terms of study materials, experimental models, interventions, and outcome measures. PubMed/MEDLINE, Web of Science Core Collection, China National Knowledge Infrastructure (CNKI), and Wanfang Data were searched from database inception to 2026. No restriction was applied to the year of publication. Publications in English or Chinese were considered.

Two complementary searches were conducted. The first search was designed to identify studies directly examining pigeonpea leaf-derived materials in orthopedic diseases or bone- and cartilage-related models. The second search was designed to identify pharmacological studies addressing prespecified mechanisms relevant to orthopedic disorders, including osteogenesis, osteoclastogenesis, angiogenesis, endothelial injury, inflammation, oxidative stress, apoptosis, and lipid metabolism. The search terms covered the botanical source, pigeonpea leaf-derived preparations, and major reported metabolites. No study-design filter was applied because the available evidence included *in vitro*, animal, and human studies. The complete database-specific search strategies are presented in Supplementary Table S1. The reference lists of the included studies and relevant reviews were also manually examined to identify additional potentially eligible studies. Taxonomic nomenclature was verified against Plants of the World Online, which recognizes *C. cajan (L.)* Huth [Fabaceae] as the accepted name.

### 2.2 Eligibility criteria

Studies were eligible for the core evidence synthesis if they: (1) investigated botanically identified *C. cajan* leaves, a pigeonpea leaf extract or fraction, a purified metabolite reported to originate from pigeonpea leaves, or a preparation in which pigeonpea leaf was the sole botanical drug; (2) reported original *in vitro*, *in vivo*, or human data; and (3) evaluated an orthopedic disease or an outcome directly related to bone, cartilage, osteogenesis, osteoclastogenesis, angiogenesis, or bone repair. Studies using non-orthopedic models were included only as supportive mechanistic evidence when they addressed a prespecified biological process relevant to the review question; these studies were explicitly identified as indirect evidence and were not used to infer therapeutic efficacy in orthopedic diseases.

Reviews, guidelines, and general disease-mechanism articles were used only to provide background information or identify additional references and were not treated as primary pharmacological evidence. Studies were excluded if they investigated only the seeds, roots, or other non-leaf parts of C. cajan; examined an unrelated nutritional or agricultural outcome; evaluated a multi-botanical preparation from which the effect of pigeonpea leaf could not be distinguished; contained only computational predictions without experimental validation; duplicated a more complete report of the same experiment; or lacked sufficient information to determine the material, model, or relevant outcome. Conference abstracts without an accessible full report were excluded. Dissertations were eligible when they contained sufficient methodological and outcome information, but they were identified as non-peer-reviewed evidence.

### 2.3 Study selection

All records in the retrieved records were combined and deduplicated using a hierarchical procedure. Duplicate records were first identified by exact DOI or PMID matching, followed by exact normalized-title matching with concordant publication year and bibliographic information. Records with highly similar titles were manually reviewed, whereas reports with conflicting DOIs, authors, publication years, sources, or publication types were retained separately. The supplied yielded 7,188 records: PubMed (n = 633), Web of Science Core Collection (n = 3,224), CNKI (n = 270), and Wanfang Data (n = 2,996). After removing 1,218 duplicate records within databases and search sets and 720 duplicates across databases, 5,250 unique records remained for title and abstract screening. Following screening and full-text assessment, 27 primary reports were included in the core evidence synthesis and appraised in Supplementary Table S2; additional publications were used only for taxonomic, background, or methodological context.

### 2.4 Data extraction and narrative synthesis

For each included primary study, the following information was extracted where available: botanical material and plant part, authentication or voucher information, type of extract, fraction, purified metabolite, or preparation, experimental model, sample size, dose or concentration, route of administration, treatment duration, controls, principal outcomes, and reported methodological safeguards such as randomization and blinding. Studies were grouped according to the orthopedic condition, the material tested, and the experimental level. Because of substantial heterogeneity in the study materials, models, interventions, and outcomes, the evidence was synthesized narratively rather than quantitatively.

## 3 Evidence categorization and critical appraisal

Because the included studies were heterogeneous in test material, experimental model, exposure, and outcome, no quantitative synthesis was attempted. Primary evidence was classified according to its proximity to the clinical question as: (1) human intervention studies; (2) disease-relevant *in vivo* studies; (3) disease-relevant *in vitro* or *ex vivo* studies; and (4) indirect or hypothesis-generating studies, including non-orthopedic cell models, medicated-serum experiments, omics analyses, and *in silico* studies. Separately, the investigated materials were categorized as crude extracts, enriched fractions, purified metabolites, or finished preparations. Findings obtained with one material category were not attributed to another unless a direct compositional or pharmacological link had been demonstrated. This hierarchy represents translational proximity and should not be interpreted as a formal GRADE certainty rating.

For each primary study, we extracted, where available, the botanical identity and plant part, authentication or voucher information, extraction and standardization procedures or purified-metabolite identity and purity, experimental model, sample size, dose or concentration range, lowest tested dose or concentration associated with a reported effect, route of administration, treatment duration, negative or vehicle controls, positive controls, principal outcomes, randomization and blinding procedures, and pharmacokinetic or safety information. Interpretability was appraised qualitatively according to material identity, exposure plausibility, control adequacy, model relevance, risk-of-bias safeguards, outcome validity, and independent replication. Information unavailable in the original publication was recorded as not reported (NR) rather than inferred. When only a single dose was tested, the minimum active dose or concentration was considered undetermined.

## 4 Major reported metabolites and pharmacological evidence for pigeonpea leaves

Pigeonpea leaves contain chemically diverse metabolites, among which flavonoids, stilbenes, phenolic acids and triterpenoids are the most frequently reported classes. Modern studies have shown that pigeonpea leaf-derived materials exert multiple biological effects, including anti-inflammatory and antioxidant activities, improvement of hemorheological properties, regulation of lipid metabolism and modulation of bone metabolism. These findings provide experimental evidence supporting their potential application in orthopedic diseases (Wang et al., 2025; Monrroy and García, 2025; Hou et al., 2024; Sun et al., 2019).

The studies included in this review used different forms of pigeonpea leaf-derived materials. These included crude pigeonpea leaf extracts, total-flavonoid fractions and stilbene-rich extracts, purified metabolites such as cajanin and cajaninstilbene acid, and Tongluo Shenggu Capsule, which contains pigeonpea leaf as its sole botanical drug. Because these materials differ in composition, results obtained with one form cannot simply be attributed to another form, particularly to a single purified metabolite.

Flavonoids represent one of the major classes of bioactive metabolites in pigeonpea leaves. Previous studies suggest that total flavonoids from pigeonpea leaves may influence bone tissue repair, angiogenesis, and inflammatory responses, and have shown potential therapeutic effects in animal models of femoral head osteonecrosis and bone defect repair (Luo et al., 2009; Li X. et al., 2020). Stilbene metabolites are mainly associated with the regulation of lipid metabolism, osteogenic–adipogenic differentiation balance and estrogen-like activity, and have attracted increasing attention in studies of steroid-induced osteonecrosis of the femoral head and osteoporosis (Zheng et al., 2007a; Luo, 2008; Liu et al., 2008). In addition, cajanin, a representative purified metabolite of pigeonpea leaves, has been reported to be involved in the regulation of apoptosis-related processes, gene expression and bone metabolism (Sun, 2018).

At present, information on the bioavailability and pharmacokinetics of pigeonpea leaf-derived extracts, fractions, and purified metabolites remains limited. Data on long-term safety are also insufficient. Therefore, the findings summarized in the following sections should be interpreted according to the specific material and experimental model used.

## 5 Pigeonpea leaf-derived preparations in orthopedic diseases

The evidence reviewed here includes published clinical studies as well as a larger body of preclinical mechanistic research. Clinical studies have evaluated Tongluo Shenggu Capsule in patients with osteonecrosis of the femoral head. These studies provide human evidence for this specific preparation and indication, but their findings should be interpreted in light of each study’s design, comparator, treatment duration, clinical outcomes, and safety reporting. Clinical observations with the finished preparation cannot be attributed directly to a particular pigeonpea leaf metabolite or extrapolated to differently prepared extracts, fractions, or other orthopedic diseases. Disease-relevant animal studies are interpreted as preclinical findings, while cell and *ex vivo* studies provide mechanistic plausibility. Findings from non-orthopedic cell models, medicated-serum experiments, omics analyses, and computational predictions are treated as indirect or hypothesis-generating evidence.

### 5.1 Osteonecrosis of the femoral head

Osteonecrosis of the femoral head (ONFH), also called avascular necrosis of the femoral head, is primarily associated with reduced blood supply to the femoral head. Once the blood supply is disturbed, osteocytes, bone marrow stromal cells and bone marrow adipocytes can be affected. As the disease progresses, the femoral head may lose its normal structure and eventually collapse. ONFH is more common in young and middle-aged adults, particularly those aged approximately 30–50 years. Long-term glucocorticoid use and heavy alcohol intake are two important risk factors (Zhou et al., 2026; Liu et al., 2024; Shao et al., 2024).

Current treatments include core decompression, bone grafting, osteotomy and total hip arthroplasty. For young patients, however, the key challenge remains delaying femoral head collapse and preserving hip function for as long as possible. For this reason, drugs that can improve local blood circulation, regulate bone metabolism and support repair of necrotic bone have received more attention in recent years.

From the perspective of TCM, ONFH is often linked to liver and kidney deficiency, phlegm and blood stasis blocking the collaterals, impaired circulation of qi and blood, and insufficient nourishment of tendons and bones. Pigeonpea leaves and related preparations have traditionally been used to promote blood circulation, remove blood stasis, reduce swelling, unblock collaterals and relieve pain. Modern studies suggest that these effects may be related to the regulation of lipid metabolism, improvement of blood flow properties, promotion of new blood vessel formation, reduction of inflammation and enhancement of osteogenic differentiation. These findings are also, to some extent, consistent with the TCM understanding of ONFH as a disorder involving “stasis, deficiency and phlegm” (Zu et al., 2023; Zhang and Yan, 2025; Wei and Hou, 2019; Zhao et al., 2020; Wang et al., 2021; Liu et al., 2021; Chen et al., 2016).

#### 5.1.1 Regulation of lipid metabolism and the osteogenic–adipogenic differentiation balance

Dysregulated lipid metabolism is an important pathological contributor to steroid- and alcohol-induced osteonecrosis of the femoral head. Previous studies have shown that patients with alcohol- or steroid-associated osteonecrosis often present with varying degrees of dyslipidemia. Elevated serum lipid levels may promote adipocyte hypertrophy, increase intraosseous pressure within the bone marrow cavity, and induce fat embolism in the microvasculature, thereby impairing local blood perfusion of the femoral head (Li et al., 2024; Zhang et al., 2024; Konarski et al., 2023; Huang et al., 2021). Therefore, serum lipid profiling may serve as a supportive indicator when assessing risk factors for osteonecrosis of the femoral head.

A stilbene-rich pigeonpea leaf fraction reduced serum lipid indices in hyperlipidemic mice (Luo, 2008). Because this model did not reproduce osteonecrosis of the femoral head, the study provides evidence of a lipid-modulating effect only and does not demonstrate the prevention or treatment of ONFH. In a separate rat BMSC study, Tongluo Shenggu Capsule altered outcomes associated with osteogenic and adipogenic differentiation (Liu et al., 2008). This finding is relevant to marrow-cell fate but cannot establish preservation of femoral-head structure or function. Moreover, these studies investigated chemically and pharmacologically different materials. Without comparable chemical characterization and exposure data, their findings cannot be attributed to a single common active metabolite or interpreted as evidence of a unified mechanism.

These effects may be related, at least in part, to the 17β-estradiol-like activity of stilbene metabolites from pigeonpea leaves. Previous studies have shown that these metabolites can activate estrogen receptors, enhance alkaline phosphatase activity, improve trabecular bone architecture, and upregulate the expression of osteogenesis-related genes, including core-binding factor α1/runt-related transcription factor 2 (Cbfα1/RUNX2), parathyroid hormone/parathyroid hormone-related peptide receptor, and osteocalcin. At the same time, they can downregulate adipogenesis-related genes, including peroxisome proliferator-activated receptor γ, fatty acid-binding protein, and lipoprotein lipase, thereby reducing adipocyte formation (Zheng et al., 2007a; Liu et al., 2008). Collectively, these findings suggest that pigeonpea leaf-derived materials may contribute to the modulation of steroid-induced osteonecrosis of the femoral head by regulating lipid metabolism and directing bone marrow mesenchymal stem cell fate toward osteogenesis rather than adipogenesis.

#### 5.1.2 Improvement of local blood circulation and promotion of angiogenesis

The development of osteonecrosis of the femoral head is closely associated with impaired local blood circulation. According to the intravascular coagulation hypothesis, glucocorticoid exposure, alcohol consumption, and other risk factors may induce a hypercoagulable state, sluggish blood flow, and vascular endothelial injury within the femoral head. These changes can establish a vicious cycle of ischemia, thrombosis, and further ischemia, ultimately leading to hypoxia, ischemic injury, and necrosis of bone cells (Ma et al., 2024).

Animal studies have shown that pigeonpea leaf extracts can reduce whole-blood viscosity and prolong prothrombin time, activated partial thromboplastin time, and thrombin time in rats with steroid-induced osteonecrosis of the femoral head. These findings suggest that pigeonpea leaf extracts may help ameliorate abnormal hemorheological and coagulation states and potentially reduce thrombosis-related risk (Zhao et al., 2020). In addition, total flavonoids from pigeonpea leaves have been reported to increase the number of vascular endothelial growth factor-positive cells in necrotic regions, promote local angiogenesis and vascular network formation, and thereby create a more favorable microenvironment for repair of necrotic bone (Luo et al., 2009). More direct evidence was provided by Yang et al., who evaluated Tongluo Shenggu Capsule in a methylprednisolone-induced rat model of glucocorticoid-induced osteonecrosis of the femoral head, together with complementary *ex vivo* and *in vitro* experiments. LC–MS analysis identified 33 metabolites in the preparation. In the rat model, micro-CT and histological examinations indicated attenuation of femoral-head damage, whereas angiography and CD31 immunohistochemistry suggested increased vascularization. Tongluo Shenggu Capsule also promoted microvessel outgrowth in rat aortic rings and improved the migration, invasion, and tube-formation capacities of dexamethasone-injured HUVECs. These effects were accompanied by increased expression of VEGF, VEGFR2, PKC, Raf-1, p-MEK, and p-ERK, supporting the involvement of the VEGF–VEGFR2–PKC–Raf-1–MEK–ERK signaling axis in its pro-angiogenic activity (Yang et al., 2023).

Further studies have shown that Tongluo Shenggu Capsule can increase the expression of phosphorylated mitogen-activated protein kinase (p-MEK) and phosphorylated extracellular signal-regulated kinase (p-ERK) in human umbilical vein endothelial cells, activate the MEK/ERK signaling pathway, and upregulate vascular endothelial growth factor receptor 2 (VEGFR2) expression, thereby protecting endothelial cells from glucocorticoid-induced injury (Wang et al., 2021). Meanwhile, glucocorticoids can induce endothelial cells to secrete endothelin-1 (ET-1), a potent vasoconstrictor, while suppressing nitric oxide production, resulting in vasoconstriction and reduced local perfusion. Tongluo Shenggu Capsule may alleviate vascular spasm and improve local ischemia of the femoral head by restoring the dynamic balance between ET-1 and nitric oxide (NO) (Wang et al., 2021). Collectively, these findings suggest that pigeonpea leaves and related preparations may contribute to the repair of osteonecrosis of the femoral head by improving hemorheological properties, protecting vascular endothelial function, and promoting angiogenesis. Although HUVEC and aortic-ring assays provide complementary evidence of pro-angiogenic activity (Wang et al., 2021), neither model reproduces the intraosseous vascular environment or the mechanical and ischemic characteristics of ONFH. These findings therefore support angiogenic plausibility but do not establish restoration of femoral-head perfusion or prevention of structural collapse. Interpretation also depends on whether the tested material was chemically standardized and whether the concentrations used were achievable *in vivo*.

#### 5.1.3 Suppression of inflammation and apoptosis

Inflammation and apoptosis play important roles in the progression of femoral head osteonecrosis. Studies have shown that the signal transducer and activator of transcription 1 (STAT1)/caspase-3 pathway is involved in osteocyte apoptosis associated with steroid-induced osteonecrosis of the femoral head. In addition, glucocorticoid-induced osteonecrosis has been linked to activation of TLR4 signaling and increased osteoclast activity (Xu et al., 2014; Tian et al., 2014).

In rats with steroid-induced ONFH, Tongluo Shenggu Capsule was reported to modify bone-remodelling-associated outcomes in femoral-head tissue (Liu et al., 2021). This disease-relevant *in vivo* study provides a preclinical efficacy signal, although its reliability should be interpreted in relation to material standardization, dose selection, comparator treatment, sample size, and the reporting of randomization and blinded outcome assessment. Separately, capsule-containing serum reduced osteoclast differentiation-associated outcomes in RANKL/M-CSF-treated RAW264.7 cells (Fan et al., 2021). The medicated-serum design does not define which metabolites reached the cells or their actual concentrations and therefore cannot establish a direct effect of the capsule or of a particular pigeonpea leaf metabolite. Cajaninstilbene acid also reduced corticosterone-associated injury in PC12 neuronal cells (Jiang et al., 2014; Liu et al., 2014). However, PC12 cells do not represent osteocytes, osteoblasts, endothelial cells of the femoral head, or bone-marrow stromal cells. These results indicate a general cytoprotective signal under the tested conditions but provide only indirect evidence for ONFH-related anti-apoptotic activity. Confirmation in disease-relevant bone and vascular cells, followed by *in vivo* target-validation experiments, is required.

#### 5.1.4 Regulation of genes related to bone repair

With the development of transcriptomic and proteomic technologies, increasing attention has been paid to the regulatory effects of pigeonpea leaves and their derived materials on gene expression in osteonecrosis of the femoral head. Gene expression profiling and related experimental studies have suggested that ATP-binding cassette subfamily B member 1 (ABCB1), RUNX2, VEGF, lysine demethylase 5D (KDM5D), Osterix, and nephronectin (NPNT) may be involved in the development and progression of steroid-induced osteonecrosis of the femoral head (Zhang et al., 2022; Zhou et al., 2015; Ma et al., 2010). Among these genes, RUNX2 and Osterix are key transcription factors involved in osteogenic differentiation (Liu et al., 2020; Komori, 2019), VEGF is primarily associated with angiogenesis, and PPARγ is closely related to adipogenic differentiation of bone marrow mesenchymal stem cells (Duffy et al., 2013; Duan et al., 2022).

Cajaninstilbene acid has been reported to modulate the protein expression of several osteonecrosis-related genes in a steroid-induced ONFH rat model. In addition, Tongluo Shenggu Capsule was shown to upregulate ABCB1 and RUNX2 at both the mRNA and protein levels, while downregulating PPARγ mRNA expression in a rabbit model of glucocorticoid-induced ONFH. These findings suggest that pigeonpea leaf-derived preparations may influence femoral head osteonecrosis by promoting osteogenic differentiation, suppressing adipogenic differentiation, and alleviating glucocorticoid-related injury (Sun, 2018; Chen et al., 2016). Liu et al. further suggested that pigeonpea leaves may affect osteogenic differentiation and necrotic bone repair through mitogen-activated protein kinase (MAPK)-related pathways (Liu et al., 2008). Proteomic analysis of glucocorticoid-treated BMSCs exposed to medicated serum prepared from a pigeonpea leaf-containing preparation identified changes in HSP- and COP9-related proteins (Ma et al., 2013).

The transcriptomic and proteomic findings identify associations between treatment and changes in gene or protein expression but do not by themselves establish direct molecular targets or causal mechanisms. In addition, pathway enrichment may be influenced by threshold selection, database coverage, and multiple-testing procedures. These findings should therefore be regarded as hypothesis-generating until confirmed by targeted protein-level measurements, pathway inhibition or knockdown experiments, rescue experiments, and replication using chemically standardized material.

Overall, studies on pigeonpea leaves and related preparations in osteonecrosis of the femoral head are relatively numerous compared with those in other orthopedic conditions. Their potential mechanisms may involve regulation of lipid metabolism, improvement of microcirculation, promotion of angiogenesis, suppression of inflammation and apoptosis, and enhancement of osteogenesis. However, most available evidence is still derived from basic experimental studies, whereas clinical investigations remain limited. Their therapeutic efficacy and translational value therefore require further validation in well-designed clinical studies. The proposed relationships among these pathways are summarized in Figure 1.

**FIGURE 1:**
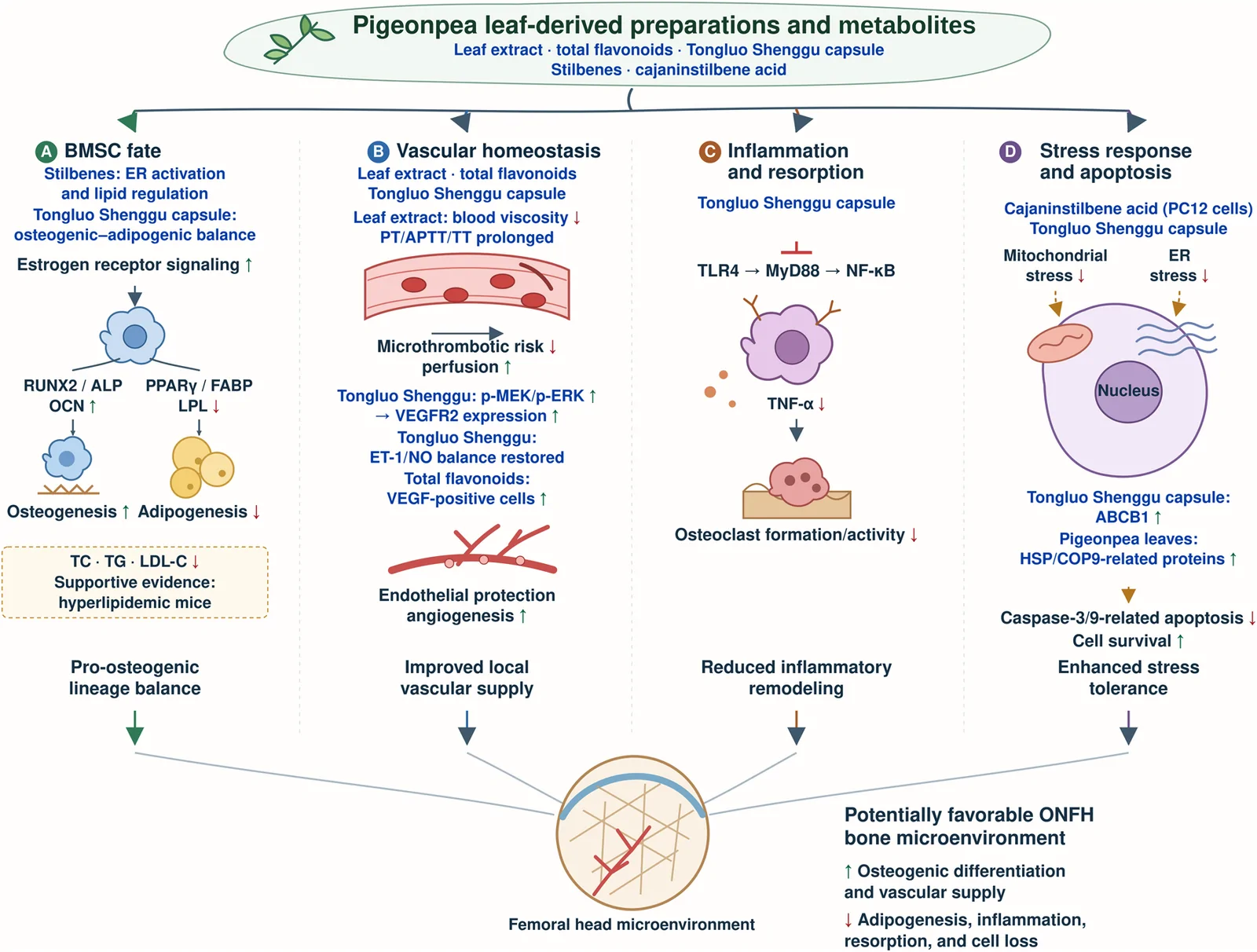
Proposed mechanisms by which pigeonpea leaf-derived materials may attenuate osteonecrosis of the femoral head (ONFH) progression. The proposed effects include regulation of lipid metabolism and osteogenic–adipogenic differentiation, improvement of microcirculation and angiogenesis, suppression of inflammatory signaling, and regulation of bone repair-related genes. Anti-apoptotic effects of cajaninstilbene acid have been reported mainly in corticosterone-induced PC12 cell injury models and have not yet been directly confirmed in osteocytes or ONFH models.

### 5.2 Osteoporosis

Osteoporosis, a metabolic bone disorder, is defined by diminished bone density, compromised microarchitectural integrity, and enhanced fragility, with these abnormalities jointly predisposing patients to a higher incidence of fractures. Its pathogenesis is complex and involves multiple factors, including genetic susceptibility, hormonal changes, nutritional deficiency, insufficient mechanical loading, and oxidative stress. The central pathological feature of osteoporosis is disruption of the dynamic balance between bone formation and bone resorption, which manifests as relatively increased osteoclast-mediated bone resorption and/or insufficient osteoblast-mediated bone formation (Zhang et al., 2025).

From the perspective of TCM, osteoporosis is generally categorized under “bone atrophy” and “bone impediment” and is considered to be associated with liver and kidney deficiency, spleen and kidney insufficiency, inadequate nourishment of qi and blood, and blood stasis obstructing the collaterals (Ge et al., 2020). Pigeonpea leaves and their bioactive metabolites have shown potential regulatory effects on bone metabolism. Their effects in osteoporosis appear to involve two main aspects: promotion of osteogenic differentiation and inhibition of osteoclast-mediated bone resorption.

#### 5.2.1 Promotion of osteoblast differentiation and bone formation

Studies have shown that Tongluo Shenggu Capsule can increase serum alkaline phosphatase levels and improve trabecular area, thickness, and number in rat models of stress-deprivation osteoporosis, suggesting a potential protective effect against bone loss associated with insufficient mechanical loading (Liu et al., 2011). However, because stress-deprivation osteoporosis differs pathophysiologically from other forms of osteoporosis, such as postmenopausal osteoporosis, the generalizability of these findings requires further validation.

At the cellular level, cajanin has been reported to regulate osteogenic differentiation in skeletal stem-cell models (Ma, 2024). In a separate study, cajanine increased mineral deposition and the expression of RUNX2, osteopontin, and collagen I in cultured human bone marrow mesenchymal stem cells (Zhao et al., 2019). RUNX2 is a key transcription factor involved in osteoblast differentiation, whereas osteopontin and collagen I contribute to bone-matrix formation and mineralization (Liu et al., 2020; Komori, 2019; Huang et al., 2007). These observations provide a mechanistic basis for osteogenic activity, but isolated cell systems do not reproduce the endocrine, immune, vascular, and mechanical environment of osteoporotic bone. Moreover, increased expression of osteogenic markers does not by itself establish improvements in bone mass, microarchitecture, mechanical strength, or fracture resistance.

The effects of cajanine on human bone marrow mesenchymal stem cell proliferation were assessed using an MTT assay at concentrations of 1, 10, and 100 ng/mL over 3–15 days (Zhao et al., 2019). Although increased MTT signals were observed, tetrazolium reduction primarily reflects cellular metabolic activity rather than directly measuring cell number and may be influenced by the optical or redox properties of plant-derived metabolites. Consequently, the proliferative effect remains to be confirmed using orthogonal measurements such as direct cell counting, EdU or BrdU incorporation, or DNA quantification. By contrast, von Kossa staining and the assessment of RUNX2, osteopontin, and collagen I provide independent support for an effect on osteogenic differentiation (Zhao et al., 2019). Nevertheless, these *in vitro* findings cannot establish anti-osteoporotic efficacy without corresponding evidence from disease-relevant animal models.

The biological material also requires careful distinction. Ref. Zhao et al. (2019) used commercially sourced cajanine rather than an extract prepared from taxonomically authenticated pigeonpea leaves. The findings therefore characterize the activity of the tested metabolite under defined culture conditions but do not directly demonstrate the osteogenic activity of pigeonpea leaves or a standardized pigeonpea leaf preparation.

#### 5.2.2 Inhibition of osteoclast-mediated bone resorption

Cajaninstilbene acid inhibited RANKL-induced osteoclast differentiation and bone-resorption activity *in vitro* and attenuated bone loss in ovariectomized mice (Sun et al., 2019). Mechanistically, these effects were associated with modulation of the MAPK, NF-κB, and Ca^2+^/NFATc1 signaling pathways. Cajaninstilbene acid altered the phosphorylation of ERK, JNK, and p38, reduced c-Fos expression, delayed IκBα degradation, and suppressed RANKL-induced Ca^2+^ oscillations and reactive oxygen species production. These signaling changes converged on NFATc1, a central regulator of osteoclast differentiation, and were accompanied by reduced expression of osteoclast-associated markers (Sun et al., 2019).

The combination of RANKL-induced osteoclast assays and an ovariectomized mouse model provides disease-relevant preclinical evidence for an antiresorptive effect. However, the relationship between the active concentrations identified *in vitro* and the systemic or bone-tissue exposure achieved *in vivo* remains unresolved. Differences in metabolite purity, dose, administration route, treatment duration, and experimental controls may also affect the reproducibility and translational relevance of the findings. Furthermore, the ovariectomized model primarily reflects estrogen-deficiency-related bone loss and does not encompass the full biological heterogeneity of human osteoporosis.

Cajanin was subsequently reported to disrupt the Siglec15/NFATc1 signaling cascade, suppress osteoclast fusion and actin-ring formation, and reduce bone-resorption activity (Ma et al., 2026). The associated preservation of trabecular bone in ovariectomized mice extends these observations beyond isolated osteoclast cultures. Nevertheless, the findings remain preclinical, and it is unclear whether comparable exposure can be achieved safely in humans. The use of the same ovariectomy-based disease context also limits extrapolation to age-related, glucocorticoid-induced, or secondary osteoporosis.

Stilbene-rich pigeonpea extracts have also been investigated in estrogen-deficiency-related bone loss. In ovariectomized rats, a stilbene-rich extract was associated with changes in bone-related and endocrine outcomes, including parathyroid hormone, calcium handling, and gonadotropin levels (Zheng et al., 2007a). A separate cell-based study examined the effects of a Cajanus cajan extract on human TE85 osteoblast-like cells and the differentiation of osteoclast-like cells (Zheng et al., 2007b). These studies examined chemically complex extracts rather than purified metabolites, and their effects cannot be attributed to a single stilbene without adequate chemical characterization and activity-guided validation.

The reported estrogen-like activity may contribute to the effects observed in ovariectomized animals, but changes in endocrine markers do not necessarily indicate a favorable clinical estrogenic effect. Estrogen-like activity may also raise tissue-selectivity and reproductive-safety concerns; endocrine-related findings should therefore not be interpreted as uniformly beneficial without appropriate uterotrophic, reproductive, and long-term safety assessments.

Across different studies, pigeonpea leaf-derived materials were associated separately with osteogenic and anti-osteoclastic outcomes. This apparent convergence is biologically relevant but does not demonstrate that one standardized intervention simultaneously produces both effects. The evidence is heterogeneous with respect to the investigated material, model, dose, and outcome, and most mechanistic observations were obtained *in vitro*.

Collectively, the current evidence supports osteogenic and antiresorptive biological plausibility together with selected preclinical effects, rather than established efficacy in the prevention or treatment of osteoporosis. Further investigation will require taxonomically authenticated and chemically standardized materials, exposure-informed dose–response designs, appropriate controls, and validation across complementary osteoporosis models. Pharmacokinetic studies, long-term safety assessments, and well-designed clinical investigations are required to determine whether these experimental effects are reproducible and clinically relevant.

### 5.3 Osteoarthritis

Osteoarthritis, a prevalent degenerative joint disorder, is marked by multiple pathological changes: gradual loss of articular cartilage, remodeling of subchondral bone, formation of osteophytes, and inflammation of the synovium. These alterations commonly lead to joint pain and compromised function. The knee, hip, and hand joints are most commonly affected. The pathogenesis of osteoarthritis is closely associated with reduced chondrocyte viability, inflammatory responses, oxidative stress, and degradation of the cartilage extracellular matrix (Tang et al., 2025).

From the perspective of TCM, osteoarthritis is generally categorized under “bone impediment” and “impediment syndrome” and is commonly associated with liver and kidney deficiency, invasion of wind, cold, and dampness, phlegm and blood stasis obstructing the collaterals, and inadequate nourishment of tendons and bones (Chen, 2020). Pigeonpea leaves have traditionally been used to resolve blood stasis, reduce swelling, and relieve pain. Current evidence concerning pigeonpea leaf-derived preparations in osteoarthritis is limited to chemically induced OA, cytokine- or oxidant-challenged cell systems, and acute cartilage-defect models (Li Y. et al., 2020; Tang et al., 2010; Lin et al., 2020).

Tongluo Shenggu Capsule was evaluated in a monosodium iodoacetate (MIA)-induced OA model in Sprague–Dawley rats and in IL-1β-challenged SW1353 cells (Li Y. et al., 2020). In the animal experiment, intra-articular injection of MIA at 3 mg per rat was followed, beginning 3 days after model induction, by treatment with Tongluo Shenggu Capsule at 0.5, 1, or 2 g/kg for 3 weeks. All three doses reduced serum IL-1β levels and the expression of MMP-13 and c-Fos in articular cartilage, with evidence of a dose-dependent response. In the cell experiment, SW1353 cells were exposed to Tongluo Shenggu Capsule at 1–100 μg/mL for 48 h and subsequently challenged with IL-1β at 20 ng/mL for 8 h. Concentrations of 10–100 μg/mL increased cell viability and modulated the expression of Wnt4α, β-catenin, GSK-3β, MMP-13, ADAMTS4, Nrf2, GCLC, and NQO-1 (Li Y. et al., 2020). These findings support anti-inflammatory and cartilage-catabolism-modulating effects under the tested conditions. However, MIA induces rapid chemical injury to articular cartilage, whereas SW1353 is a human chondrosarcoma-derived cell line. Neither model fully reproduces the chronic, mechanically influenced, and multicellular pathology of human OA.

Tongluo Shenggu Capsule was also reported to promote repair in a rabbit model of full-thickness knee articular cartilage defects (Tang et al., 2010). This model examines the repair of an acutely created focal cartilage lesion rather than the progressive degeneration characteristic of OA. The findings therefore support a potential effect on cartilage repair but do not demonstrate disease-modifying activity in chronic OA.

The antioxidant effects of Tongluo Shenggu Capsule were investigated in SW1353 cells pretreated with 1, 5, 10, 50, or 100 μg/mL for 72 h before exposure to H_2_O_2_ at 300 μmol/L for 1 h (Lin et al., 2020). Tongluo Shenggu Capsule increased cell viability, enhanced Nrf2 and HO-1 expression, and reduced Keap1 protein expression. These observations support an antioxidant and cytoprotective response in an acute oxidant-injury model. Changes in Wnt4α and β-catenin were also reported, although the available data do not establish whether Wnt/β-catenin signaling is causally required for Nrf2/HO-1 activation (Wu et al., 2023; Lian et al., 2020). Moreover, acute H_2_O_2_ exposure does not reproduce the sustained inflammatory, biomechanical, and extracellular-matrix changes associated with OA, and the use of a chondrosarcoma-derived cell line further limits direct extrapolation to primary human articular chondrocytes.

Evidence for individual purified metabolites of pigeonpea leaves in OA is more indirect. Cajaninstilbene acid and several synthesized derivatives reduced the production of nitric oxide, TNF-α, and IL-6 in LPS-stimulated macrophages and modulated neutrophil and macrophage migration in an injured zebrafish model (Huang et al., 2016). These effects were associated with NF-κB, MAPK, and PPARγ-related signaling. However, neither the macrophage assays nor the zebrafish injury model directly assessed articular chondrocytes or OA progression. The findings therefore demonstrate general anti-inflammatory activity rather than direct cartilage protection or disease modification in OA. Furthermore, effects observed with individual synthetic derivatives should not be attributed uniformly to the parent metabolite cajaninstilbene acid.

Collectively, the available studies provide preclinical signals of cartilage-protective and inflammation-modulating activity. However, the evidence derives from heterogeneous models, including chemically induced OA, acute cartilage defects, oxidant-injured chondrocytes, and non-chondrocytic inflammatory systems. These models address different biological questions and should not be regarded as interchangeable. In particular, an acute cartilage-defect model does not fully reproduce the chronic, mechanically driven, and multifactorial pathology of human OA. No conclusion regarding clinical symptom relief or disease modification can presently be drawn.

### 5.4 Bone defect repair

Bone defects refer to pathological conditions characterized by disruption of bone integrity and localized loss of bone tissue. They may result from trauma, infection, tumor resection, congenital developmental abnormalities, or other causes. Large bone defects can impair skeletal support and motor function and, in severe cases, may lead to limb deformity and functional disability. The repair of bone defects requires coordinated contributions from multiple biological and material-related factors, including osteoblasts, bone marrow mesenchymal stem cells, angiogenesis, the inflammatory microenvironment, and scaffold materials (Xue et al., 2022; Yu et al., 2025; Lv et al., 2024; Su et al., 2022).

Pigeonpea leaves and their major metabolites have been reported to promote osteogenic differentiation, inhibit osteoclast formation, and modulate local inflammatory responses, suggesting potential value in bone defect repair. However, direct evidence in this field remains limited. Existing studies mainly reflect the broader strategy of combining metabolites from TCM with biomaterials to enhance bone tissue repair, rather than using pigeonpea leaf-derived materials alone as pharmacological agents for bone defects.

Li et al. used an α-calcium sulfate hemihydrate scaffold loaded with total flavonoids from pigeonpea leaves in a rat calvarial defect model. Compared with the scaffold alone, the flavonoid-loaded composite promoted new bone formation (Li X. et al., 2020). This comparison suggests that the addition of the total-flavonoid fraction enhanced the bone-repair effect of the scaffold. However, the tested intervention was the composite scaffold as a whole, and the observed effect cannot be attributed solely to the total-flavonoid fraction. The scaffold itself may influence osteoconduction, local retention and release, and surrounding tissue responses. Moreover, the rat calvarial defect is a non-load-bearing model and differs substantially from the mechanical and biological environment of long-bone nonunion or large segmental defects. Thus, the study supports the use of the total-flavonoid fraction as a local scaffold additive but does not establish its efficacy following oral administration or in the clinical treatment of bone defects.

Bone defect repair requires both new bone formation and adequate vascularization. The osteogenic and angiogenic effects reported for pigeonpea leaf-derived materials in other orthopedic models may therefore also be relevant to bone regeneration. However, these effects have not yet been directly confirmed in bone defect models. The study using total flavonoids combined with an α-calcium sulfate hemihydrate scaffold suggests that pigeonpea leaf-derived materials may be useful as bioactive additives to bone-repair materials. Further studies are needed to determine whether similar effects can be reproduced using standardized extracts or purified metabolites and whether they are associated with enhanced osteogenesis and vascularization.

Overall, research on pigeonpea leaf-derived materials in bone defect repair remains at an early stage. Further studies should address the chemical characterization and quality control of the total-flavonoid fraction, its release profile and local exposure, scaffold biocompatibility, long-term degradation and mechanical performance, and efficacy in load-bearing critical-size defect models.

### 5.5 Integrated mechanistic framework of pigeonpea leaf-derived materials in orthopedic diseases

Although the signaling pathways described above were identified in different experimental models, they appear to converge on several closely related biological processes involved in bone and joint tissue repair. At the level of bone metabolism, pigeonpea leaf-derived materials may promote osteogenic differentiation by increasing the expression of RUNX2, Osterix, ALP, OCN, and OPN, while reducing adipogenic differentiation through regulation of PPARγ. At the same time, cajaninstilbene acid suppresses RANKL/RANK-mediated MAPK, NF-κB, and Ca^2+^/NFATc1 signaling, thereby inhibiting osteoclast formation and bone resorption. These effects suggest that pigeonpea-derived materials may contribute to the maintenance of bone remodeling by acting on both bone formation and bone resorption.

These effects are also associated with changes in the vascular and inflammatory microenvironment. Activation of MEK/ERK and VEGFR2 signaling, together with regulation of the ET-1/NO balance, may improve endothelial function and local blood supply, providing favorable conditions for bone repair. Meanwhile, inhibition of TLR4/MyD88/NF-κB signaling may reduce inflammatory responses, whereas activation of the Nrf2/ARE pathway enhances antioxidant defense and protects cells against oxidative injury. In osteoarthritis-related studies, changes in Wnt/β-catenin signaling occur together with activation of Nrf2/ARE and reduced cartilage matrix degradation, suggesting that oxidative stress and cartilage-degrading pathways may be functionally linked rather than acting independently. Together, these findings suggest that the effects of pigeonpea leaf-derived materials may result from coordinated regulation of bone metabolism, vascular function, inflammation, oxidative stress, and cell survival, thereby creating a microenvironment more favorable for tissue repair. However, direct interactions among these pathways have not yet been fully demonstrated and require further experimental verification.

## 6 Assay interference and PAINS considerations

Several polyphenolic metabolites discussed in this review may behave as pan-assay interference compounds (PAINS) under particular assay conditions. Activity detected only by molecular docking, in a single *in vitro* assay, or at high concentrations may result from colloidal aggregation, redox or optical interference, covalent reactivity, or nonspecific membrane perturbation rather than selective target engagement (Magalhães et al., 2021; Bolz et al., 2021). However, a PAINS alert does not by itself establish inactivity, because the underlying mechanisms and binding modes vary across structural classes (Bolz et al., 2021). Such findings are therefore considered hypothesis-generating unless supported by concentration-dependent effects at non-cytotoxic concentrations, appropriate interference controls or counterscreens, orthogonal assays, and confirmation in disease-relevant models.

## 7 Conclusions and future perspectives

As a traditional medicinal resource, pigeonpea leaves and their bioactive metabolites have been associated with bone-, vascular-, inflammatory- and oxidative-stress-related outcomes in osteonecrosis of the femoral head, osteoporosis, osteoarthritis, and bone defect repair. Available experimental evidence suggests that these effects are mainly associated with the regulation of bone metabolism, improvement of microcirculation and angiogenesis, and attenuation of inflammation, oxidative stress, and apoptosis. These pharmacological actions are broadly consistent with the traditional use of pigeonpea leaves in promoting blood circulation and unblocking meridians, reducing swelling, and strengthening bones and tendons.

However, the current evidence base remains incomplete. Although experimental studies on pigeonpea leaves in orthopedic diseases have increased in recent years, several important research gaps remain. These mainly involve the identification and standardization of active metabolites, clarification of key molecular mechanisms, validation in disease-specific models, and the translation of preclinical findings into clinical studies. The pharmacodynamic basis of pigeonpea leaves has not been fully clarified. The molecular targets underlying the reported bone-related effects also require further investigation. More importantly, a substantial translational gap remains between the available experimental findings and their clinical application. In addition, most existing studies are based on cellular or animal models, whereas clinical evidence is still limited. Tongluo Shenggu Capsule, whose sole botanical drug is pigeonpea leaf, has been widely used in experimental studies and provides evidence for the potential bone-protective effects of pigeonpea leaves. However, differences in extract preparation, dosage, and experimental models may affect the comparability of these studies. In addition, studies using standardized pigeonpea leaf extracts or purified metabolites such as cajaninstilbene acid and cajanin remain relatively limited. Pharmacokinetic data, dose-response studies, and long-term safety evaluations are also limited. Therefore, whether the effects observed in experimental studies can be reproduced in clinical practice remains unclear. Further studies are therefore needed to confirm these findings.

Another important gap concerns fracture nonunion. Although pigeonpea leaf-derived materials have been associated with osteogenic, angiogenic, anti-inflammatory, and bone-repair-related outcomes in experimental studies of osteonecrosis, osteoporosis, and bone defects, their direct effects in fracture nonunion have rarely been investigated. Because nonunion involves a complex pathological process that includes impaired osteogenesis, inadequate vascularization, persistent inflammation, possible infection, and mechanical instability, findings from osteogenic differentiation or bone defect repair models cannot be directly applied to nonunion.

Future studies could focus on better-characterized materials, including total-flavonoid fractions, stilbene-rich extracts, and purified metabolites such as cajanin. These materials could be examined in disease-relevant models of osteonecrosis of the femoral head, osteoporosis, osteoarthritis, and bone-defect repair to determine which biological processes are affected. Existing studies have suggested that pigeonpea leaf-related materials may affect processes such as osteogenic differentiation, osteoclast formation, vascular endothelial injury, and oxidative stress in chondrocytes. Future studies could continue to examine changes in indicators such as RUNX2, Osterix, RANKL/NFATc1, VEGF, TLR4/NF-κB, Nrf2/HO-1, and Wnt/β-catenin. Researchers could also use gene silencing, pathway inhibitors, and animal experiments to further determine whether there is a clear association between these indicators and the pharmacological effects of these materials. Multi-omics approaches may also help identify changes in genes, proteins, and metabolites associated with the effects of pigeonpea leaf-derived materials. Network pharmacology and computational analyses may be used to generate hypotheses concerning metabolite–target relationships, but these predictions require experimental confirmation.

Future studies should also pay greater attention to the clinical translation of these experimental findings. The pharmacokinetic characteristics, appropriate dose ranges, long-term safety, and clinical efficacy of standardized pigeonpea leaf preparations need to be further investigated before their role in orthopedic diseases can be properly evaluated. Emerging approaches such as cell- and gene-based therapies are also being explored for bone and joint disorders. At present, however, their therapeutic development differs substantially from that of pigeonpea leaf-derived pharmacological interventions, and direct comparison is not supported by available evidence. Any potential complementary relationship would therefore require direct experimental evaluation.

In summary, pigeonpea leaves and related preparations remain of interest in preclinical orthopedic research, particularly in conditions involving bone metabolic imbalance, ischemic bone injury, and bone tissue repair. Nevertheless, most of this evidence remains preclinical, and randomized clinical trials, pharmacokinetic studies, dose-response investigations, and long-term safety data are still limited. The clinical value of pigeonpea leaves and their bioactive metabolites therefore remains to be confirmed by further translational and clinical studies. With continued advances in both basic and clinical research, pigeonpea leaves may become a valuable focus for research on TCM-based strategies for orthopedic disease management. The available evidence supports further preclinical and translational evaluation but does not yet establish the clinical efficacy of pigeonpea leaf-derived materials in orthopedic disorders.
