# Supplementary material for: Pigeonpea leaves in orthopedic diseases: bioactive metabolites, pharmacological actions and therapeutic potential
Source: Front Pharmacol. 2026 Oct 7;17:1918539. doi: 10.3389/fphar.2026.1918539 (PMC13683585; doi:10.3389/fphar.2026.1918539)
Supplement: Supplementary file 1 [file DataSheet1.docx]

Supplementary Table S1

**Database-specific search strategies used in this narrative review**

**PubMed/MEDLINE**

| **Search ID** | **Search focus and field** | **Exact search strategy** | **Coverage and final search date** | **Records retrieved** |
| --- | --- | --- | --- | --- |
| P1 | Botanical source, leaf-derived preparations, and reported metabolites (Title/Abstract) | "Cajanus cajan"[Title/Abstract] OR pigeonpea[Title/Abstract] OR "pigeon pea"[Title/Abstract] OR "pigeonpea leaf"[Title/Abstract] OR "pigeonpea leaves"[Title/Abstract] OR "Folium Cajani"[Title/Abstract] OR "Cajani folium"[Title/Abstract] OR "Tongluo Shenggu"[Title/Abstract] OR "Tongluo Shenggu capsule"[Title/Abstract] OR Tongluoshenggu[Title/Abstract] OR "Fufang Shengmai Chenggu"[Title/Abstract] OR cajanin[Title/Abstract] OR cajanine[Title/Abstract] OR "cajaninstilbene acid"[Title/Abstract] | — | — |
| P2 | Phytochemistry, pharmacokinetics, and safety terms (Title/Abstract) | leaf[Title/Abstract] OR leaves[Title/Abstract] OR phytochem*[Title/Abstract] OR metabolite*[Title/Abstract] OR constituent*[Title/Abstract] OR flavonoid*[Title/Abstract] OR stilbene*[Title/Abstract] OR "phenolic acid"[Title/Abstract] OR "phenolic acids"[Title/Abstract] OR triterpenoid*[Title/Abstract] OR chromatograph*[Title/Abstract] OR HPLC[Title/Abstract] OR "LC-MS"[Title/Abstract] OR pharmacokinetic*[Title/Abstract] OR bioavailab*[Title/Abstract] OR safety[Title/Abstract] OR toxic*[Title/Abstract] | — | — |
| P3 | Orthopedic diseases and pharmacological mechanisms (Title/Abstract) | osteonecrosis[Title/Abstract] OR ONFH[Title/Abstract] OR "osteonecrosis of the femoral head"[Title/Abstract] OR "femoral head necrosis"[Title/Abstract] OR "necrosis of the femoral head"[Title/Abstract] OR "avascular necrosis"[Title/Abstract] OR osteoporosis[Title/Abstract] OR osteoarthritis[Title/Abstract] OR "bone defect"[Title/Abstract] OR "bone defects"[Title/Abstract] OR "bone regeneration"[Title/Abstract] OR "bone repair"[Title/Abstract] OR "bone tissue engineering"[Title/Abstract] OR "fracture healing"[Title/Abstract] OR nonunion[Title/Abstract] OR "bone metabolism"[Title/Abstract] OR osteogen*[Title/Abstract] OR osteoblast*[Title/Abstract] OR osteoclast*[Title/Abstract] OR osteoclastogen*[Title/Abstract] OR adipogen*[Title/Abstract] OR cartilage[Title/Abstract] OR chondrocyte*[Title/Abstract] OR chondrogen*[Title/Abstract] OR "bone marrow mesenchymal stem cell"[Title/Abstract] OR "bone marrow mesenchymal stem cells"[Title/Abstract] OR "skeletal stem cell"[Title/Abstract] OR "skeletal stem cells"[Title/Abstract] OR angiogen*[Title/Abstract] OR endothelial[Title/Abstract] OR microcirculation[Title/Abstract] OR hemorheolog*[Title/Abstract] OR coagulation[Title/Abstract] OR "lipid metabolism"[Title/Abstract] OR inflamm*[Title/Abstract] OR antioxidant*[Title/Abstract] OR "oxidative stress"[Title/Abstract] OR apoptosis[Title/Abstract] OR apoptotic[Title/Abstract] OR RANKL[Title/Abstract] OR NFATc1[Title/Abstract] OR RUNX2[Title/Abstract] OR VEGF[Title/Abstract] OR TLR4[Title/Abstract] OR "NF-kappa B"[Title/Abstract] OR Nrf2[Title/Abstract] OR "Wnt/beta-catenin"[Title/Abstract] OR MAPK[Title/Abstract] OR "MEK/ERK"[Title/Abstract] OR PPARgamma[Title/Abstract] | — | — |
| **P4** | **Final phytochemistry, pharmacokinetics, and safety search** | P1 AND P2 | Inception to 2026 | n = 460 |
| **P5** | **Final orthopedic diseases and pharmacological mechanisms search** | P1 AND P3 | Inception to 2026 | n = 173 |

**Web of Science Core Collection**

| **Search ID** | **Search focus and field** | **Exact search strategy** | **Coverage and final search date** | **Records retrieved** |
| --- | --- | --- | --- | --- |
| W1 | Botanical source, leaf-derived preparations, and reported metabolites (Topic) | TS=("Cajanus cajan" OR pigeonpea OR "pigeon pea" OR "pigeonpea leaf" OR "pigeonpea leaves" OR "Folium Cajani" OR "Cajani folium" OR "Tongluo Shenggu" OR "Tongluo Shenggu capsule" OR Tongluoshenggu OR "Fufang Shengmai Chenggu" OR cajanin OR cajanine OR "cajaninstilbene acid") | — | — |
| W2 | Phytochemistry, pharmacokinetics, and safety terms (Topic) | TS=(leaf OR leaves OR phytochem* OR metabolite* OR constituent* OR flavonoid* OR stilbene* OR "phenolic acid*" OR triterpenoid* OR chromatograph* OR HPLC OR "LC-MS" OR pharmacokinetic* OR bioavailab* OR safety OR toxic*) | — | — |
| W3 | Orthopedic diseases and pharmacological mechanisms (Topic) | TS=(osteonecrosis OR ONFH OR "osteonecrosis of the femoral head" OR "femoral head necrosis" OR "necrosis of the femoral head" OR "avascular necrosis" OR osteoporosis OR osteoarthritis OR "bone defect*" OR "bone regeneration" OR "bone repair" OR "bone tissue engineering" OR "fracture healing" OR nonunion OR "bone metabolism" OR osteogen* OR osteoblast* OR osteoclast* OR osteoclastogen* OR adipogen* OR cartilage OR chondrocyte* OR chondrogen* OR "bone marrow mesenchymal stem cell*" OR "skeletal stem cell*" OR angiogen* OR endothelial OR microcirculation OR hemorheolog* OR coagulation OR "lipid metabolism" OR inflamm* OR antioxidant* OR "oxidative stress" OR apoptosis OR apoptotic OR RANKL OR NFATc1 OR RUNX2 OR VEGF OR TLR4 OR "NF-kappa B" OR Nrf2 OR "Wnt/beta-catenin" OR MAPK OR "MEK/ERK" OR PPARgamma) | — | — |
| **W4** | **Final phytochemistry, pharmacokinetics, and safety search** | W1 AND W2 | Inception to 2026 | n = 2636 |
| **W5** | **Final orthopedic diseases and pharmacological mechanisms search** | W1 AND W3 | Inception to 2026 | n = 588 |

**CNKI**

| **Search ID** | **Search focus and field** | **Exact search strategy** | **Coverage and final search date** | **Records retrieved** |
| --- | --- | --- | --- | --- |
| C1 | Botanical source, leaf-derived preparations, and reported metabolites (Subject) | 木豆 OR 木豆叶 OR 鸽豆 OR 树豆 OR 木豆总黄酮 OR 木豆叶总黄酮 OR 木豆芪类 OR 木豆叶芪类提取物 OR 木豆芪酸 OR 木豆素 OR 木豆异黄酮 OR 通络生骨 OR 通络生骨胶囊 OR 复方生脉成骨胶囊 OR Cajanus cajan OR cajanin OR cajanine OR cajaninstilbene acid | — | — |
| C2 | Phytochemistry, pharmacokinetics, and safety terms (Subject) | 化学成分 OR 植物化学 OR 代谢物 OR 黄酮 OR 总黄酮 OR 芪类 OR 二苯乙烯类 OR 酚酸 OR 三萜 OR 指纹图谱 OR 色谱 OR 高效液相色谱 OR 液相色谱-质谱 OR 药代动力学 OR 生物利用度 OR 安全性 OR 毒性 | — | — |
| C3 | Orthopedic diseases and pharmacological mechanisms (Subject) | 股骨头坏死 OR 股骨头缺血性坏死 OR 糖皮质激素性股骨头坏死 OR 骨坏死 OR 骨质疏松 OR 骨关节炎 OR 软骨损伤 OR 软骨缺损 OR 骨缺损 OR 骨缺陷 OR 骨修复 OR 骨再生 OR 骨组织工程 OR 骨折愈合 OR 骨不连 OR 骨代谢 OR 成骨 OR 成骨细胞 OR 破骨细胞 OR 破骨细胞形成 OR 成脂分化 OR 骨髓间充质干细胞 OR 血管生成 OR 血管内皮 OR 微循环 OR 血液流变学 OR 凝血 OR 脂质代谢 OR 炎症 OR 抗氧化 OR 氧化应激 OR 凋亡 OR RANKL OR NFATc1 OR RUNX2 OR VEGF OR TLR4 OR NF-κB OR Nrf2 OR Wnt/β-catenin OR MAPK OR MEK/ERK OR PPARγ | — | — |
| **C4** | **Final phytochemistry, pharmacokinetics, and safety search** | C1 AND C2 | Inception to 2026 | n = 153 |
| **C5** | **Final orthopedic diseases and pharmacological mechanisms search** | C1 AND C3 | Inception to 2026 | n = 179 |

**Wanfang Data**

| **Search ID** | **Search focus and field** | **Exact search strategy** | **Coverage and final search date** | **Records retrieved** |
| --- | --- | --- | --- | --- |
| F1 | Botanical source, leaf-derived preparations, and reported metabolites (Subject) | 木豆 OR 木豆叶 OR 鸽豆 OR 树豆 OR 木豆总黄酮 OR 木豆叶总黄酮 OR 木豆芪类 OR 木豆叶芪类提取物 OR 木豆芪酸 OR 木豆素 OR 木豆异黄酮 OR 通络生骨 OR 通络生骨胶囊 OR 复方生脉成骨胶囊 OR Cajanus cajan OR cajanin OR cajanine OR cajaninstilbene acid | — | — |
| F2 | Phytochemistry, pharmacokinetics, and safety terms (Subject) | 化学成分 OR 植物化学 OR 代谢物 OR 黄酮 OR 总黄酮 OR 芪类 OR 二苯乙烯类 OR 酚酸 OR 三萜 OR 指纹图谱 OR 色谱 OR 高效液相色谱 OR 液相色谱-质谱 OR 药代动力学 OR 生物利用度 OR 安全性 OR 毒性 | — | — |
| F3 | Orthopedic diseases and pharmacological mechanisms (Subject) | 股骨头坏死 OR 股骨头缺血性坏死 OR 糖皮质激素性股骨头坏死 OR 骨坏死 OR 骨质疏松 OR 骨关节炎 OR 软骨损伤 OR 软骨缺损 OR 骨缺损 OR 骨缺陷 OR 骨修复 OR 骨再生 OR 骨组织工程 OR 骨折愈合 OR 骨不连 OR 骨代谢 OR 成骨 OR 成骨细胞 OR 破骨细胞 OR 破骨细胞形成 OR 成脂分化 OR 骨髓间充质干细胞 OR 血管生成 OR 血管内皮 OR 微循环 OR 血液流变学 OR 凝血 OR 脂质代谢 OR 炎症 OR 抗氧化 OR 氧化应激 OR 凋亡 OR RANKL OR NFATc1 OR RUNX2 OR VEGF OR TLR4 OR NF-κB OR Nrf2 OR Wnt/β-catenin OR MAPK OR MEK/ERK OR PPARγ | — | — |
| **F4** | **Final phytochemistry, pharmacokinetics, and safety search** | F1 AND F2 | Inception to 2026 | n = 837 |
| **F5** | **Final orthopedic diseases and pharmacological mechanisms search** | F1 AND F3 | Inception to 2026 | n = 2159 |
