# Supplementary material for: Pigeonpea leaves in orthopedic diseases: bioactive metabolites, pharmacological actions and therapeutic potential
Source: Front Pharmacol. 2026 Oct 7;17:1918539. doi: 10.3389/fphar.2026.1918539 (PMC13683585; doi:10.3389/fphar.2026.1918539)
Supplement: Supplementary file 2 [file DataSheet2.docx]

**Supplementary Table S2. Individual-study evidence extraction and methodological appraisal of pigeonpea leaf-related orthopedic studies**

**Definitions.**  ONFH, osteonecrosis of the femoral head; OA, osteoarthritis; OVX, ovariectomy; MIA, monosodium iodoacetate; BMSC, bone-marrow mesenchymal stem cell; HUVEC, human umbilical-vein endothelial cell; CSA, cajaninstilbene acid; PT, prothrombin time; APTT, activated partial thromboplastin time; TT, thrombin time.

**Material definition.** The accepted taxon is *Cajanus cajan (L.)* Huth [Fabaceae]. Tongluo Shenggu Capsule is treated as a monobotanical pigeonpea-leaf preparation rather than a polyherbal formula. Material appraisal considered the plant part, taxonomic authentication/voucher specimen, extraction method, chemical standardization or purity, supplier, and batch; missing items are identified in the relevant row.

**Appraisal framework.** Human studies, disease-relevant animal studies, orthopedic cell/ex vivo studies and indirect models were interpreted separately. ‘Fewer concerns’, ‘Some concerns’ and ‘Major concerns’ are transparent narrative judgments based on model relevance, material definition, dose/exposure reporting, controls, sample size, randomization/blinding, outcome validity and reproducibility; they are not a validated numerical risk-of-bias score.

**Assay-interference caution.** For polyphenols and stilbenes, activity confined to a single in vitro assay, particularly at high concentration, was interpreted cautiously because aggregation, redox or optical interference and other promiscuous effects may produce false-positive signals. A PAINS alert was treated as a reason for orthogonal confirmation, not as automatic evidence of inactivity [67,68].

| **Ref.** | **Evidence type** | **Material and characterization** | **Model / sample** | **Dose, route and duration; lowest active exposure** | **Negative / positive controls** | **Reported measured endpoints** | **Critical appraisal (evidence basis; overall concern)** |
| --- | --- | --- | --- | --- | --- | --- | --- |
| **[10]** | Human clinical report | Tongluo Shenggu Capsule, a single-botanical preparation containing pigeonpea leaf; the botanical drug was described as the dried leaves and tender twigs of Cajanus cajan. Each capsule contained 0.5 g. Plant authentication, voucher specimen, manufacturing method, batch information and chemical standardization were not reported. | Randomized, double-blind, double-dummy, active-controlled multicenter clinical trial involving 214 patients with ARCO stage II–III ONFH (treatment, n = 108; control, n = 106), aged 18–65 years. | Oral administration of four capsules per dose (0.5 g/capsule), three times daily for 3 months (6 g/day). Only one dose was tested; the lowest effective dose was not established. | Jiangu Sheng Wan was used as the active comparator (4.5 g per dose, three times daily) with a double-dummy design. No placebo-only or untreated control was included. Both groups received the same functional-exercise and load-reduction instructions. | Clinical symptom and sign scores, radiographic grading of necrotic-bone resorption and new-bone formation, adverse events and laboratory safety indices. Clinical response rates were 84.26% versus 81.13% (P = 0.2933), whereas radiographic outcomes favored Tongluo Shenggu Capsule. | The randomized, double-blind, multicenter design strengthens the evidence. However, product standardization, allocation concealment, sample-size calculation, participant attrition and trial registration were not reported. The symptom score was investigator-defined, no placebo-only group was included, and the 3-month follow-up was insufficient to assess structural progression or long-term safety. A nonsignificant difference from the active comparator does not establish equivalence or non-inferiority. Overall concern: Major. |
| **[11]** | Human clinical report | Tongluo Shenggu Capsule, also termed Compound Shengmai Chenggu Capsule by the authors. Pigeonpea leaf was described as the principal botanical drug. The complete composition, equivalence of the two product names, plant authentication, extraction method, manufacturer, batch and chemical standardization were not reported. | Prospective, single-center, uncontrolled clinical study involving 142 patients (216 affected hips). The cohort included 98 men and 44 women, aged 12–70 years, with ARCO stage I–IV ONFH. Ethics approval and informed consent were reported. | Four capsules orally, three times daily. One treatment course lasted 3 months, and eight courses (24 months) were reported. Capsule strength and the lowest effective dose were not reported. | No active comparator, placebo or untreated control group was included. | Clinical symptoms, Harris score, hip-preservation efficacy score, ARCO stage, categorical clinical response and adverse events. The reported overall response rate was 90.00%; Harris and hip-preservation scores improved after treatment. No withdrawals, loss to follow-up or serious adverse events were reported. | This uncontrolled before–after study provides limited clinical evidence and cannot distinguish treatment effects from natural disease variation or other influences. Product composition and standardization were inadequately reported. The stated inclusion age of 18–60 years conflicts with the enrolled age range of 12–70 years, and the reported 24-month treatment period is difficult to reconcile with the recruitment and publication dates. Analyses also mixed patient- and hip-level data without accounting for bilateral hips, and some reported test statistics and P values appear inconsistent. Overall concern: Major. |
| **[12]** | Disease-relevant animal study | Pigeonpea leaf total-flavonoid fraction. The botanical source, authentication, voucher specimen, extraction method, flavonoid content, chemical markers, purity, manufacturer and batch were not reported | Sixty SD rats (200 ± 20 g; both sexes) were randomly divided into six groups (n = 10/group). ONFH was induced in the left femoral head by repeated liquid-nitrogen freezing. | Total flavonoids were injected intramuscularly around the left hip at 20, 40 or 80 mg/kg once daily for 4 weeks. The lowest tested dose was 20 mg/kg, but the minimum effective dose was not established. | Sham-operated and model-control groups received saline. Compound Xiangdan Injection (0.1 mL/300 g) served as the positive control. | Serial radiographs, gross femoral-head appearance, HE histology, new-bone formation and VEGF immunohistochemistry. Qualitative improvements were reported in all treatment groups, with the greatest changes at 80 mg/kg. | The study included sham, model and positive controls and tested three doses. However, the flavonoid fraction was not chemically characterized, and randomization procedures, blinding, sample-size calculation and animal ethics approval were not reported. Outcomes were mainly qualitative, without adequate quantitative analysis or statistical comparisons. The acute cryonecrosis model and local injection route do not reproduce the chronic pathogenesis or clinical administration of human ONFH. Overall concern: Major. |
| **[24]** | Disease-relevant animal study | Commercial Tongluo Shenggu Capsule, obtained from Zhejiang Hisun Pharmaceutical, was used as the pigeonpea leaf preparation. The batch, capsule content, extraction method, chemical profile and marker-based standardization were not reported. | Fifty male SD rats were randomized into normal-control, model-control and three dose groups (n = 10/group). Steroid-associated ONFH was induced using lipopolysaccharide followed by methylprednisolone. Third-passage BMSCs and medicated serum obtained from the rats were used for the subsequent cell experiment. | Tongluo Shenggu Capsule suspension was administered by oral gavage at 0.48, 0.96 or 1.44 g/kg once daily for 4 weeks. The lowest tested dose showing activity was 0.48 g/kg/day; the minimum effective dose was not established. BMSCs were treated with medicated serum for 1 week, but the serum concentration was not reported. | Normal and ONFH model controls were included; no positive-drug control was used. The cell experiment included an untreated blank group but no positive control. | Whole-blood and plasma viscosity, PT, APTT, TT and femoral-head HE histology were assessed in vivo. BMSC outcomes included TG, ALP, osteocalcin, and mineralized-nodule area. Dose-related improvements were reported. | Random allocation, three dose levels and quantitative hemorheological outcomes strengthen the study. However, the preparation was not chemically standardized, and blinding, sample-size calculation and animal ethics approval were not reported. Treatment began during model induction, so the findings mainly indicate preventive or early-stage effects rather than treatment of established ONFH. Histological assessment was qualitative, and the medicated-serum experiment lacked a reported serum concentration and adequate comparator groups. Overall concern: Major. |
| **[26]** | Disease-relevant animal study | Tongluo Shenggu Capsule administered as an aqueous suspension; product name was reported, whereas marker-based standardization and botanical voucher were not reported. | Steroid-induced ONFH in rats; normal, model and treatment groups (n = 15 per group). Reporting of allocation concealment and blinded outcome assessment was not identified. | 0.4 g kg⁻¹ by oral gavage once daily for 4 weeks; single dose only, so a minimum active dose was not established. | Normal and model groups received matched saline; no pharmacological positive control. | Femoral-head histology and bone-remodelling-associated findings; TLR4, MyD88, NF-κB and inflammatory markers. | **Overall concern: Some.** The disease-relevant model and matched model control support a preclinical signal, but the single-dose design, absence of an active comparator and limited reporting of blinding and product standardization restrict causal and translational interpretation. |
| **[27]** | Disease-relevant animal study | Commercial Tongluo Shenggu Capsule from Zhejiang Hisun Pharmaceutical (batch 661404), prepared as an aqueous suspension. Its botanical composition, extraction method and chemical standardization were not reported. | Twenty New Zealand rabbits were randomized into normal (n = 6), model (n = 7) and treatment (n = 7) groups. ONFH was induced using LPS followed by six methylprednisolone injections. Two modelled rabbits died, leaving six animals in each modelled group. | Tongluo Shenggu Capsule was administered orally at 0.3 g/kg once daily for 16 weeks, beginning 24 h after the final methylprednisolone injection. Only one dose was tested; the minimum effective dose was not established. | Normal and ONFH model controls received an equal volume of vehicle. No positive-drug control was included. | MRI, HE histology, ONFH incidence and mRNA/protein expression of ABCB1, RUNX2, OPN and PPARγ. ONFH was reported in 4/6 model animals and 2/6 treated animals; molecular analyses used three samples per group. | The randomized, disease-relevant rabbit model and molecular measurements support mechanistic exploration. However, treatment began before ONFH was confirmed, so the study mainly assessed prevention or early intervention rather than repair of established ONFH. MRI and histology were not quantitatively or blindly assessed, and molecular analyses used only three samples per group. Importantly, the reported difference in ONFH incidence (4/6 versus 2/6) does not remain significant using Fisher’s exact test (P = 0.567), contrary to the reported P < 0.05. Sample-size calculation, animal ethics approval and chemical standardization were not reported. Overall concern: Major. |
| **[33]** | Animal + ex vivo + vascular-cell study | Tongluo Shenggu Capsule (TLSGC), a single-botanical preparation made from the water extract of pigeonpea leaves [Cajanus cajan (L.) Huth, Fabaceae], supplied by Zhejiang Hisun Pharmaceutical Co., Ltd.; approval no. Z20040001. LC-MS tentatively characterized 33 metabolites. Batch number, extraction yield or drug-extract ratio, quantitative marker content, voucher specimen and raw-material authentication were not reported. The article used the outdated botanical citation Cajanus cajan (Linn.) Millsp. | A total of 144 Sprague-Dawley rats (72 male and 72 female; 300–320 g) were allocated by random-number table to six groups (n=24/group). GIONFH was induced with LPS and methylprednisolone. Reported endpoint analyses generally used n=8/group, whereas pathway assays used n=3/group. Dex-inhibited HUVECs (passages 4–8) and rat aortic rings were also examined (n=3/condition). | Rats received TLSGC at 0.6, 1.2 or 2.4 g/kg once daily by gavage from day 6 for 3 months. GIONFH was induced by LPS 10 μg/kg via tail-vein injection for 2 days, followed by methylprednisolone 21 mg/kg intramuscularly for 3 days. Ex vivo and cell experiments used TLSGC at 12.5, 25 and 50 ng/mL with Dex 0.5 μg/mL; aortic rings were cultured for 5 days, migration/invasion for 12 h and tube formation for 2 h. The MTT assay tested 3.125–400 ng/mL for 24 h. The lowest tested exposure associated with activity in selected endpoints was 0.6 g/kg in rats and 12.5 ng/mL in cell/ex vivo assays, although effects were not consistent across all endpoints. | Animal controls comprised a normal group and a GIONFH model group receiving double-distilled water. Henggu Gushangyu Heji (HGGSY; 5.3 mL/kg every 2 days) was used as a positive treatment control. Cell and aortic-ring experiments included untreated controls, Dex-only model controls and VEGF 20 ng/mL as a positive control. | Qualitative LC-MS profiling; femoral-head Micro-CT parameters (BMD, BV/TV, Tb.Th, Tb.N, Tb.Sp and BS/BV); HE histology, empty lacunae and adipocyte accumulation; Microfil and ink angiography; CD31 staining; hemorheology, coagulation, lipid, NO and ET-1 indices; aortic-ring sprouting; HUVEC migration, invasion, tube formation and viability; VEGF/VEGFR2 expression and PKC, Raf-1, MEK/ERK phosphorylation. | Random allocation, three TLSGC doses, positive controls and concordant animal, ex vivo and cell outcomes strengthen the preclinical evidence. However, the selection of n=8 or n=3 from the original n=24/group was not explained, and blinding and sample-size calculation were not reported. The preparation was characterized qualitatively but not quantitatively standardized, and pharmacokinetic or tissue-exposure data were unavailable. Network analysis is hypothesis-generating, while increased VEGF-pathway protein expression does not establish pathway causality in the absence of receptor blockade, pathway inhibition or genetic intervention. MTT results were not shown, although the additional functional assays provide orthogonal support. The study supports preclinical angiogenic and structural effects but does not establish clinical efficacy. Overall concern: Some concerns. |
| **[16]** | Orthopedic cell study | Commercial Tongluo Shenggu Capsule (Hisun Pharmaceutical; approval No. Z20040001), described as a single-botanical preparation derived from pigeonpea leaf. A dry-extract solution (0.5 g/10 mL water) was used. Batch number, extraction procedure, botanical authentication and chemical standardization were not reported. | Third-passage bone marrow stromal cells isolated from female rat femurs and cultured under dexamethasone-induced osteogenic/adipogenic differentiation conditions. Six replicates per group were reported, but their biological independence was unclear. | Direct in vitro exposure for 10 days. The dry-extract solution was added at 2.5, 5 or 10 mL per 55 mL culture medium. Activity was reported at the lowest tested dose, but the exact final concentrations and minimum active concentration were not clearly established. | The bidirectional differentiation group served as the main comparator; separate adipogenic and osteogenic differentiation groups were included. No pharmacological positive control or clearly volume-matched vehicle control was reported. | Semiquantitative RT-PCR analysis of LPL and OPN mRNA, normalized to GAPDH. Treatment was associated with reduced LPL and increased OPN expression, although the LPL results were not strictly dose-dependent. | The study provides preliminary in vitro evidence of altered lineage-associated gene expression. However, the tested extract was insufficiently characterized, and no cell-viability, protein-level or functional differentiation assays were performed. These findings do not establish efficacy in osteonecrosis or osteoporosis. Overall concern: Major. |
| **[25]** | Indirect vascular-cell study | Commercial Tongluo Shenggu Capsule powder (Zhejiang Hisun Pharmaceutical Hangzhou Co., Ltd.; batch No. 20181130), described as a pigeonpea leaf-derived aqueous preparation. A 50 mg/mL stock was prepared in PBS and filtered. Chemical fingerprint, marker content and botanical authentication were not reported. | Ex vivo thoracic aortic rings from SD rats and passage 4–8 HUVECs exposed to methylprednisolone sodium succinate (MPS) and VEGF. Replicates varied by assay (n=3–6); rat characteristics and the independence of replicates were not reported. | Direct exposure to TLSGC at 12.5, 25 and 50 μg/L. Aortic rings were treated for 5 days; HUVEC assays lasted 3–24 h depending on the endpoint. The lowest reported active concentration was 12.5 μg/L. | Untreated, VEGF-stimulated and VEGF + MPS model groups were included. No established pharmacological positive control was used. | Aortic-ring microvessel number, length and area; HUVEC migration, invasion and tube formation; NO and ET-1 levels; and VEGFR2, p-MEK/MEK and p-ERK/ERK protein expression. TLSGC partially reversed the MPS-associated changes in these outcomes. | Multiple angiogenesis-related endpoints were assessed across ex vivo and cellular models. However, the preparation was not chemically standardized, concentration units were reported inconsistently, and supporting viability data were not presented. No pathway-inhibition experiment was conducted to establish MEK/ERK dependence. The findings provide indirect mechanistic evidence rather than evidence of efficacy in ONFH. Overall concern: Major. |
| **[36]** | Medicated-serum cell study | Tongluo Shenggu Capsule, described as containing pigeonpea leaf alone and obtained from the hospital pharmacy. Manufacturer, batch number, extraction procedure, botanical authentication and chemical standardization were not reported. | RANKL/M-CSF-induced differentiation of RAW264.7 cells. Drug-containing serum was prepared from 28 female C57BL/6 mice (12 weeks old; 18–20 g; seven per group). Animal randomization and ethics approval were reported. | Mice received TLSGC orally at 0.455, 0.91 or 1.82 g/kg/day for 14 days. Cells were treated with 20% drug-containing serum once daily for 5 days. The serum from the 0.455 g/kg/day group showed activity, but the actual cellular exposure to TLSGC metabolites was unknown. | A normal cell group and serum from saline-treated mice were included. No established anti-osteoclastic agent or TLR4/NF-κB inhibitor was used as a positive control. | CCK-8 cell viability, cell morphology, TRAP staining, TLR4 and NF-κB p65 protein expression, and TNF-α levels. Drug-containing serum was associated with reduced osteoclast formation and lower TLR4, NF-κB p65 and TNF-α outcomes. | The study included three donor-dose groups, a saline-serum control and a viability assessment. However, medicated serum does not define the active cellular exposure, and neither the capsule nor the serum was chemically characterized. The response was not consistently dose-dependent, osteoclast formation was not quantitatively assessed, and pathway dependence was not tested using an inhibitor or rescue experiment. The RANKL-induced cell model provides indirect mechanistic evidence and does not demonstrate efficacy in ONFH. Overall concern: Major. |
| **[46]** | Exploratory orthopedic-cell omics study | Yuan’s Shengmai Shenggu Tablet (Guangzhou Kangyuan Pharmaceutical Co.; batch No. 121003), described as containing pigeonpea leaf. Extraction procedure, botanical authentication, chemical fingerprint and marker content were not reported. | Third-passage human bone marrow-derived MSCs isolated from patients undergoing joint replacement for hip osteoarthritis. Donor number, demographic characteristics, MSC phenotypic identification, ethical approval and informed consent were not reported. | Female SD rats received the preparation orally at 0.6 g/kg/day for 7 days to prepare medicated serum. MSCs were exposed to 10⁻⁵ mol/L methylprednisolone for 3 days and subsequently cultured with 10% medicated serum for 5 days. Only one dose was tested; the minimum active exposure was therefore not established | Normal MSCs cultured with serum from saline-treated rats and methylprednisolone-treated MSCs cultured with control serum were included. No pharmacological positive control was used. | Two-dimensional gel electrophoresis and MALDI-TOF mass spectrometry. Methylprednisolone altered 38 protein spots, whereas 28 spots differed between the medicated-serum and model groups. Highlighted proteins included NMHC, FASN, HSP70, COP9 subunit 5 and HSP27. | This exploratory proteomic study identified candidate protein changes but did not report the number of human donors, biological replicates, statistical criteria or multiple-testing control. The proteomic findings were not independently validated, and no apoptosis, MAPK activation, osteogenic differentiation or mineralization assays were performed. Medicated serum also leaves the cellular exposure chemically undefined. The findings are hypothesis-generating and do not demonstrate osteogenic activity or ONFH repair. Overall concern: Major. |
| **[17]** | Dissertation; disease-model and transcriptomic study | Purified cajaninstilbene acid (CSA). For the cell experiments, CSA was supplied by the Institute of Medical Biotechnology, Chinese Academy of Medical Sciences; the preparation was described as involving solvent extraction, with biosynthetic preparation also mentioned. For the rat study, 300 mg synthetic CSA was supplied by Prof. Li Zhuorong’s group. Purity, batch information, voucher specimen and taxonomic authentication were not reported. The plant origin was inconsistently described as leaf/root in one section and leaf/fruit in another. | RNA-seq: 4 SLE patients with SIONFH, 4 SLE patients without SIONFH, and 4 healthy volunteers; RT-qPCR validation, 12 per group; Western blot validation, 3 per group. SVEC endothelial cells, E17.5 mouse fetal metatarsals, and patient-derived human BMSCs. Male Wistar rats were randomized to CSA, model and normal-control groups (n=10/group); endpoint-specific sample sizes were generally 3–6 per group. | Cell assays used 0–10 μM CSA for cytotoxicity testing; 10 μM was used for endothelial migration (16 h), tube formation (24 h), fetal-metatarsal angiogenesis (14 days), and BMSC osteogenic differentiation (up to 21 days). Rat study: CSA 5 mg/kg intraperitoneally every 2 days for 6 weeks (21 injections), beginning after a 5-day recovery period. SIONFH was induced with LPS 20 μg/kg intraperitoneally twice and methylprednisolone 40 mg/kg intramuscularly three times. The lowest active exposure was not established. | Human comparison groups included SLE patients without SIONFH and healthy volunteers. Endothelial assays used PBS as a negative control, bFGF or VEGF as positive controls, and dexamethasone as a disease-mimicking condition. BMSC experiments included untreated/control and dexamethasone groups; BMP-2 was used as a positive control in the mineralization assay. Rats received a normal PBS control or model PBS control. No standard pharmacological treatment comparator was reported. | PBMC RNA-seq, RT-qPCR and Western blot validation of NPNT and KDM5D; endothelial-cell migration, MTS viability, Matrigel tube formation, CD31-positive metatarsal vascular density, and ERK1/2, p38 and AKT signaling; BMSC ALP activity/staining, Alizarin red mineralization, and Wnt/β-catenin-, KDM5D-, RUNX2-, Osterix- and OPG-related proteins; rat femoral-head histology, empty lacunae, Microfil vascular density/number/length, Micro-CT parameters and protein expression of NPNT, KDM5D, VEGF, β-catenin, RUNX2, Osterix and ABCB1 | The rat experiment provides a disease-relevant preclinical signal, whereas the endothelial, metatarsal and BMSC experiments mainly support mechanistic plausibility. The human component is a small observational biomarker study and does not demonstrate treatment efficacy. Interpretation is limited by a single CSA concentration and animal dose, small endpoint-specific sample sizes, absence of pharmacokinetic data or a standard-treatment comparator, and no reported blinding. No pathway-inhibitor or rescue experiment was performed. CSA source and plant origin were inconsistently reported, and purity was not stated. Overall concern: Major concerns. |
| **[15]** | Dissertation; indirect animal study | Stilbene-containing extract fraction from pigeonpea leaves (sECC), supplied by the Institute of Medicinal Plant Development, Chinese Academy of Medical Sciences. HPLC showed three major stilbene-type metabolites at a relative ratio of 4:1:0.26. Absolute contents, extraction solvent and procedure, purity, batch information and voucher authentication were not reported. | Male Kunming mice (n=84; 19–21 g), randomly assigned to seven groups (n=12/group): normal control, hypercholesterolemic model, sECC low-dose, sECC high-dose, flavonoid extract low-dose, flavonoid extract high-dose and simvastatin groups. Hypercholesterolemia was induced by a high-fat diet containing cholesterol, sodium cholate and lard for 28 days. | sECC was administered by gavage at 100 or 200 mg/kg/day for 28 days. The 100 mg/kg/day dose reduced serum LDL-C, whereas broader lipid effects, including reductions in serum total cholesterol, triglycerides and liver lipid content, were mainly observed at 200 mg/kg/day. | A basal-diet control group and a high-fat-diet vehicle model group were included. Simvastatin (12 mg/kg/day) served as a pharmacological positive control. | Body weight, liver weight index, serum total cholesterol, triglycerides, LDL-C and HDL-C, hepatic total cholesterol and triglycerides, serum SOD activity, and hepatic HMG-CoA reductase, CYP7A1 and LDL-receptor mRNA expression. High-dose sECC reduced several serum and hepatic lipid indices and increased serum SOD activity. | The study included random allocation, an established positive control and a relatively clear dose range. However, the high-fat-diet model represents dyslipidemia rather than ONFH or another orthopedic disease, so the findings support lipid-modulating activity only. The extract was incompletely characterized, and mechanistic conclusions were based mainly on semi-quantitative mRNA measurements without pharmacokinetic, histological or vascular-function validation. Overall concern: Major. |
| **[37]** | Indirect non-orthopedic cell study | CSA isolated from pigeonpea leaves; reported purity >98%. Voucher/batch details were not identified. | PC12 neuronal cells exposed to corticosterone (100 μM for 48 h); assay n generally 3–6. | CSA 1, 2, 4, 8 or 16 μM; desipramine at matching concentrations. Lowest observed effect: 2 μM for MTT/apoptotic-nuclei outcomes and 4 μM for LDH under the reported conditions. | Untreated and corticosterone model controls; desipramine positive control. | MTT, LDH, Hoechst staining, DNA fragmentation, Ca²⁺, mitochondrial permeability/ΔΨm, cytochrome c, caspase-3 and ICAD. | **Overall concern: Some for the cellular finding, but major indirectness for ONFH.** Multiple orthogonal endpoints and a positive control reduce single-assay uncertainty, yet PC12 cells do not represent bone or femoral-head vascular cells. |
| **[38]** | Indirect non-orthopedic cell study | Cajaninstilbene acid (CSA) isolated from the leaves of *Cajanus cajan (L.)* Huth [Fabaceae] (reported as C. cajan (L.) Millsp. in the original article); purity >95% by HPLC. The isolation procedure was cited from an earlier publication. Voucher specimen, botanical authentication, collection origin and batch information were not reported. | Corticosterone-injured PC12 rat pheochromocytoma cells. Four experimental replicates were used for most assays; staining and Western blot analyses generally used three experiments. | CSA 1, 2, 4, 8 or 16 μmol/L, administered 24 h before exposure to corticosterone (200 μmol/L, 48 h). The lowest reported active concentration was 1 μmol/L in the MTT assay and 2 μmol/L for LDH release and intracellular Ca²⁺; most mechanistic assays were conducted only at 8 μmol/L. | Untreated control, corticosterone-only injury control and CSA-alone control (8 μmol/L for selected assays). DMSO was maintained below 0.1%, but a matched vehicle control was not clearly described. No established positive pharmacological control was included. | Cell viability, Hoechst 33342/PI staining, LDH release, intracellular Ca²⁺, ROS, MDA, SOD and CAT; ER morphology and expression of GRP78, GADD153/CHOP, XBP-1, caspase-12 and caspase-9. | Purified CSA was tested over a concentration range, and the MTT result was partly supported by LDH release and apoptosis staining. However, PC12 cells are a tumor-derived neuronal cell line unrelated to orthopedic disease. Most mechanistic outcomes were examined at a single concentration, and no positive control, pathway inhibition or genetic validation was included. Possible interference with colorimetric, fluorescent and redox-sensitive assays was not specifically excluded. The findings provide indirect mechanistic evidence only and do not demonstrate orthopedic efficacy. Overall concern: major. |
| **[49]** | Disease-relevant animal study (specific subtype) | Tongluo Shenggu Capsule, a single-botanical preparation derived from pigeonpea leaf extract (Zhejiang Hisun Pharmaceutical Co., Ltd.; batch 080201). Extraction procedure, chemical profile, standardization markers, botanical authentication and voucher specimen were not reported. | Forty male SPF Sprague-Dawley rats (4 weeks old, 80–100 g) were randomized to normal control, tail-suspension model, low-dose and high-dose groups (nominally n=10 per group). Tail suspension was maintained for 21 days. | Oral gavage at 0.6 or 1.2 g/kg/day for 20 days, beginning on the day after model induction. The lowest tested dose associated with significant changes in selected endpoints was 0.6 g/kg/day. | Normal non-suspended and untreated tail-suspended controls were included. Vehicle administration to the control groups was not described, and no active anti-osteoporotic comparator was used. | Serum ALP, tartrate-resistant acid phosphatase, ionized calcium and total calcium; femoral and tibial histomorphometry, including trabecular area, osteoblast and osteoclast indices, trabecular thickness, separation and number. | Random allocation, two dose levels and histomorphometric assessment provide preliminary evidence in a disuse-related bone-loss model. However, treatment was initiated during model induction and therefore evaluates prevention or attenuation rather than treatment of established osteoporosis. The preparation was not chemically characterized, and blinding, sample-size calculation, ethical approval and handling of animal losses were not reported. Approximately 15% of the animals developed tail necrosis, but their disposition was unclear. Table 3 reports n=12 despite allocation of 40 rats to four groups, whereas the other tables report n=10. The findings were not consistently dose-dependent, and several high-dose osteoclast or trabecular outcomes changed in an unfavourable direction. The tail-suspension model cannot be directly extrapolated to postmenopausal, senile or glucocorticoid-induced osteoporosis. Overall concern: major concerns. |
| **[50]** | Dissertation; cell/mechanistic study | Commercial cajanin (CA; CAS 32884-36-9; Wuhan ChemFaces Biochemical Co., Ltd.; lot CFN98419). Compound purity and independent analytical confirmation were not reported. No pigeonpea plant material was directly investigated. | RANKL-induced osteoclastogenesis in primary BMMs from male C57BL/6J mice and RAW264.7 cells; FACS-isolated mouse skeletal stem cells (mSSCs); ovariectomized C57BL/6J female mice (18 mice randomized into sham, OVX and OVX + CA groups; n=6/group). Ethics approval: 20220623003. | Osteoclast assays: CA 10-50 μmol/L for BMMs and 10-40 μmol/L for RAW264.7 cells for approximately 5 days; most mechanistic assays used 40 μmol/L. The lowest clearly reported active concentration was 20 μmol/L for selected osteoclast-related outcomes. mSSC assays: 0.25 and 0.5 μmol/L for 21 days; the lowest active concentration was 0.25 μmol/L. Animal study: 4 mg/kg CA intraperitoneally every other day for 6 weeks, beginning 3 days after ovariectomy; the minimum effective in vivo dose was not determined. | Baseline cells without RANKL and RANKL-induced model controls were included. BMP-2 (50 ng/mL) was used as a positive control in the mSSC osteogenic assay. TFAP (2.5 μmol/L), described as a PTGS1 inhibitor, was used in the mechanistic experiment. Animal controls were sham + vehicle and OVX + vehicle; no active anti-osteoporosis comparator was included. | Cell viability; TRAcP-positive osteoclast formation; F-actin rings; osteoclast-related genes and proteins; MAPK, NF-κB, Ca²⁺ and NFATc1 signaling; bulk RNA sequencing; PTGS1 expression and inhibitor experiments; mSSC mineralization and osteogenic gene expression; femoral micro-CT, HE, TRAcP, ALP and Masson staining, and PTGS1 immunofluorescence. | The study combined primary cells, a cell line, FACS-isolated mSSCs and a disease-relevant OVX model. However, compound purity was not reported, most mechanistic assays used a single concentration, and the doses used for osteogenic qPCR differed between the Methods (0.5 and 1 μmol/L) and the Results table (0.25 and 0.5 μmol/L). The animal experiment used only one dose, with no active comparator, pharmacokinetic assessment, power calculation or reported blinding. Because treatment began 3 days after ovariectomy, the study primarily addresses prevention of early bone loss rather than treatment of established osteoporosis. The proposed PTGS1 interaction was not confirmed by a direct binding assay or genetic intervention. Independent peer-reviewed replication is required. Overall concern: some concerns. |
| **[51]** | Human bone-cell mechanistic study | Commercial cajanine (Sigma-Aldrich, Shanghai); purity, batch and taxonomically authenticated leaf provenance not reported. | Human donor BMSCs, passage ≤4; donor number not reported. Cells were seeded at 3 × 10⁴ per six-well plate for osteogenic experiments. | MTT: 1, 10 and 100 ng mL⁻¹ on days 3–15; lowest MTT change at 1 ng mL⁻¹ on day 6. Osteogenic-assay concentration was not reported; 24-h pretreatment followed by approximately 16 days of induction. | Untreated/PBS and osteogenic-induction medium controls; no pharmacological positive control. | MTT, von Kossa staining, collagen I, osteopontin, RUNX2 and Rb/CDK2/cyclin B1/CDC25A/B. | **Overall concern: Major.** Donor number and osteogenic dose were absent, exposure timing was inconsistently described, and the MTT proliferation inference lacked cell-count/DNA-incorporation confirmation. Differentiation endpoints support mechanism, not anti-osteoporotic efficacy. |
| **[9]** | Disease-relevant animal + osteoclast study | Purified cajaninstilbene acid (CSA), purchased from the Institute of Botany, Chinese Academy of Sciences, and dissolved in PBS. Purity, batch number, and independent analytical confirmation were not reported. | RANKL-induced osteoclastogenesis in primary mouse bone-marrow macrophages; RAW264.7 NF-κB/NFAT reporter cells; primary mouse osteoblast cultures; ovariectomized C57BL/6 mice (sham, n=9; OVX, n=10; OVX+CSA, n=9). | CSA 1–10 μM for 5 days in osteoclastogenesis assays; 10 μM in most mechanistic assays. The lowest concentration associated with reduced osteoclast formation was 2.5 μM. In vivo, CSA was administered intraperitoneally at 10 mg/kg every 2 days for 6 weeks (21 injections), beginning 5 days after ovariectomy. | RANKL-free and RANKL-only controls in vitro; sham+PBS and OVX+PBS groups in vivo. No established antiresorptive drug was included as a positive control. | Osteoclast formation and bone resorption; osteoclast-related gene and protein expression; NF-κB/NFAT signaling, Ca²⁺ oscillations and ROS; osteoblast ALP activity and mineralization; tibial micro-CT and histomorphometry. | The concentration–response experiments, complementary osteoclast assays and randomized OVX model provide direct preclinical evidence for an anti-osteoclastic effect. CSA did not promote osteoblast differentiation in vitro. Material purity, blinding, power calculation, pharmacokinetics, in vivo dose–response and an active comparator were not reported. Treatment commenced shortly after ovariectomy and therefore reflects attenuation of early bone loss rather than treatment of established osteoporosis. Overall concern: some concerns. |
| **[53]** | Disease-relevant animal + osteoclast study | Purified cajanin (CA; purity >98%; Wuhan ChemFaces), prepared as a 50 mM stock solution in DMSO. Product batch and independent confirmation of identity or purity were not reported. No pigeonpea leaf material or extract was directly investigated. | Mouse bone marrow-derived macrophages isolated from 6–8-week-old C57BL/6J mice and stimulated with RANKL (50 ng/mL). The in vivo study used 18 eight-week-old female C57BL/6J mice randomly allocated to sham + vehicle, OVX + vehicle, and OVX + CA groups (n = 6/group). Ethical approval was reported (No. 20220623003). | CA was tested at 10–40 μM during RANKL-induced osteoclast differentiation for 5 days; selected functional and molecular assays used 20 or 40 μM, and RNA sequencing used 40 μM for 7 days. Cytotoxicity was assessed at 0–50 μM for 48 h using CCK-8. Figure 1B indicates an effect on osteoclast number at 10 μM, although the Results text identifies significant effects at 20–40 μM. OVX mice received CA at 4 mg/kg intraperitoneally every other day for 6 weeks, beginning 7 days after surgery. Only one in vivo dose was tested; therefore, the minimum effective in vivo dose was not determined. | In vitro controls included RANKL-negative cells and RANKL-treated cells without CA. Animal controls comprised sham-operated mice receiving vehicle and OVX mice receiving vehicle. No pharmacological positive-control treatment was included, and the composition of the injection vehicle was not specified. | Cell viability; TRAP-positive multinucleated osteoclast formation; osteoclast size and nuclei number; F-actin ring formation; hydroxyapatite resorption; osteoclast-related gene and protein expression; MAPK and NF-κB signaling; p65 and NFATc1 nuclear localization; and bulk RNA sequencing. In vivo outcomes included body weight, distal-femur H&E and TRAP staining, osteoclast number and surface, and micro-CT measurements of BV/TV, BS, Tb.N, and BMD. | The study used complementary in vitro assays and an OVX mouse model. However, only one in vivo dose was tested, no pharmacological positive control or exposure data were provided, and direct targeting of Siglec15 was not established. The findings support preclinical anti-osteoclastic activity of cajanin but not clinical efficacy. Overall concern: some concerns. |
| **[14]** | Disease-relevant animal study | A stilbene-rich extract of Cajanus cajan leaves (sECC). Leaves collected in Qiongshan, Hainan, were authenticated by Professor Shouquan Lin, with a specimen deposited at the Institute of Medicinal Plant Development. The leaves were extracted three times with 90% ethanol at 80°C for 1.5 h and concentrated under vacuum. Total stilbenes were standardized to 50%; the extract contained cajanin A, cajanin and cajanin C. | Sixty 3-month-old female Wistar rats: sham-operated (n=8), ovariectomized control (n=11), 17β-estradiol (n=11), and sECC at 50, 100 or 200 mg/kg (n=10 per group). | sECC was administered orally at 50, 100 or 200 mg/kg once daily, 6 days per week for 8 weeks, beginning 2 weeks after ovariectomy. The clearest endocrine and histological effects occurred at 200 mg/kg; a statistically defined minimum effective dose for bone protection was not established. | Sham-operated and untreated OVX controls received deionized water (10 mL/kg). 17β-Estradiol at 0.5 mg/kg served as the positive control. | Body and uterine weights; serum E2, FSH and LH; semiquantitative HE assessment of femoral trabecular morphology. | The authenticated material, defined extraction procedure, three-dose design and estradiol comparator strengthen the study. However, randomization, blinding, power calculation and ethical approval were not reported. Bone protection was evaluated mainly by semiquantitative histology without BMD, micro-CT, biomechanical testing or bone-turnover markers. The histological percentages were not supported by a formal between-group statistical analysis. The extract was a mixture, so its effects cannot be assigned to an individual metabolite. Lack of uterine-weight gain alone is insufficient to establish endocrine or reproductive safety. The findings provide a preliminary OVX-related signal but do not demonstrate clinical efficacy in postmenopausal osteoporosis. Overall concern: some concerns. |
| **[54]** | Orthopedic cell study | Purified cajanin and four stilbene-enriched pigeonpea leaf fractions (32-1, 35-1, 35-2 and 35-3) obtained from plants of different geographical and floral origins. The fractions were prepared by 80% ethanol reflux extraction, defatting and chromatographic separation, with total stilbenes adjusted to 50%. Cajanin was described by molecular formula, crystal appearance and melting point; HPLC purity, batch information and voucher specimens were not reported. | Human osteosarcoma-derived HOS TE85 osteoblast-like cells and osteoclast-like cells differentiated from Wistar rat bone-marrow cells using GM-CSF and 1,25(OH)₂D₃. Most assays used six wells per group; mineralization assessment used three wells. | Cajanin and the fractions were tested at 1×10⁻⁹, 1×10⁻⁸ and 1×10⁻⁷ g/mL (approximately 1, 10 and 100 ng/mL). Osteoblast-related assays lasted 48 h, mineralization 18 days and osteoclast differentiation 6 days. Significant changes were reported from 1 ng/mL for some osteoblast-associated outcomes, whereas significant inhibition of osteoclast-like cell formation was observed from 10 ng/mL for cajanin and fraction 32-1. | Culture-medium controls and 17β-estradiol at 1×10⁻⁹ mol/L were included. A vehicle-matched control was not clearly described. | MTT absorbance, ^3H-proline incorporation, ALP activity, Alizarin Red staining and numbers of TRAP-positive multinucleated osteoclast-like cells. | The use of several functional readouts and an estradiol comparator provides mechanistic information. However, HOS TE85 is a tumorl-derived cell line rather than a primary human osteoblast model. MTT absorbance does not directly measure proliferation, and possible color or redox interference from the extracts was not excluded by cell-free controls or orthogonal cell-counting methods. Mineralization was evaluated qualitatively, and osteoclast activity was not confirmed using a resorption assay. The fractions differed in source and biological activity and cannot be treated as a single standardized intervention. Numbers of independent experiments, multiple-comparison correction and material purity were insufficiently reported. The study supports in vitro mechanistic plausibility only and cannot establish an anti-osteoporotic effect. Overall concern: major concerns. |
| **[57]** | Disease-relevant animal + cartilage-cell study | Tongluo Shenggu Capsule; manufacturer/batch and quantitative chemical standardization not reported in the article. | MIA-induced knee OA in Sprague–Dawley rats (3 mg/rat); IL-1β-challenged SW1353 human chondrosarcoma cells. | Rats: 0.5, 1 or 2 g kg⁻¹ for 3 weeks, starting 3 days after MIA; all three doses affected reported outcomes. Cells: 1–100 μg mL⁻¹ for 48 h, then IL-1β 20 ng mL⁻¹ for 8 h; lowest reported cellular effect 10 μg mL⁻¹. | Normal and MIA model controls; untreated and IL-1β controls. Pharmacological positive control not reported. | Serum IL-1β; cartilage MMP-13 and c-Fos; cell viability; Wnt4α/β-catenin/GSK-3β, MMP-13, ADAMTS4, Nrf2, GCLC and NQO-1. | **Overall concern: Some.** Dose ranges and matched model controls support a preclinical signal, but MIA causes rapid chemical injury and SW1353 is tumorl derived; randomization/blinding, product standardization and an active comparator were insufficiently reported. |
| **[58]** | Adjacent animal cartilage-repair study | Tongluo Shenggu Capsule, described as a single-botanical preparation containing pigeonpea leaf extract, manufactured by Zhejiang Hisun Pharmaceutical Co., Ltd. (approval no. Z20040001; standard YBZ00012004-2007Z). Extraction procedure, batch number, chemical profile and standardization markers were not reported. | Fifty 6-month-old New Zealand rabbits (25 male and 25 female) were randomized into five groups (n=10 per group). A 4-mm-diameter and 5-mm-deep full-thickness osteochondral defect extending into the marrow cavity was created in the right femoral trochlea. | Tongluo Shenggu Capsule was administered by oral gavage at 2, 4 or 8 g/kg after surgery, with assessment at 12 weeks. The dosing frequency was not clearly specified in the methods. The lowest tested dose, 2 g/kg, was associated with a lower Wakitani score than the untreated model. | An untreated defect group served as the negative control, and Compound Danshen Tablet at 0.36 g/kg/day served as the positive comparator. No vehicle-treated control was included | Gross joint and repair-tissue appearance; HE, toluidine blue and picrosirius-red staining; scanning electron microscopy; and Wakitani histological scores. Eight specimens per group were used for histology and two for electron microscopy. | Random allocation, three dose levels, an active comparator and standardized histological scoring strengthen the study. However, blinding, sample-size calculation, ethical approval and the sex distribution within each group were not reported. The proposed proliferation and migration of mesenchymal stromal cells were inferred from morphology rather than directly measured. Mechanical properties, durability, molecular markers and longer-term outcomes were not assessed. Most importantly, this is an acute focal osteochondral-defect model and does not reproduce chronic, mechanically driven human osteoarthritis. The study supports a cartilage-repair signal but not symptomatic or disease-modifying efficacy in OA. Overall concern: some concerns. |
| **[59]** | Cartilage-cell mechanistic study | Tongluo Shenggu Capsule; batch and quantitative chemical standardization not reported in the article. | SW1353 cells exposed to H₂O₂ (300 μmol L⁻¹ for 1 h); replicate number not clearly reported. | Pretreatment with 1, 5, 10, 50 or 100 μg mL⁻¹ for 72 h; the minimum concentration producing a consistent effect across endpoints was not established. | Untreated and H₂O₂ injury controls; pharmacological antioxidant positive control not reported. | Cell viability, Nrf2, HO-1, Keap1, Wnt4α and β-catenin. | **Overall concern: Major.** Acute oxidant injury and a chondrosarcoma-derived line do not reproduce chronic OA; viability findings require orthogonal confirmation, and the data do not show that Wnt/β-catenin is causally required for Nrf2/HO-1 activation. |
| **[62]** | Indirect non-OA cell + zebrafish study | Cajaninstilbene acid (CSA) and synthetic derivatives 5b-5k were synthesized in the authors’ laboratory, with reported purity above 97% and structural identification by ^1H and ^13C NMR. The study did not directly examine pigeonpea leaf material or an extract. Compounds were dissolved in DMSO, with the final DMSO concentration fixed at 0.1%. | LPS-stimulated RAW264.7 mouse macrophages, PMA-differentiated U937 human monocyte-derived cells, and 4-day-old double-transgenic zebrafish larvae subjected to tail injury. Six to eight larvae were evaluated per group. | CSA was tested at 10, 20 and 50 μM for NO production; 20 μM was used for cytokine and pathway analyses. Cells were pretreated for 2 h before stimulation with LPS at 100 ng/mL. Zebrafish larvae received 1.25 or 5 μM CSA or derivative 5c and were assessed at 3 and 6 h. The lowest active concentrations reported were 10 μM in the cellular NO assay and 1.25 μM in the zebrafish migration assay. | Unstimulated, LPS-only, vehicle and tail-injury controls were included. Indomethacin served as the positive anti-inflammatory comparator. The PPARγ inhibitor GW9662 at 5 μM was used for mechanistic evaluation. | NO production, TNF-α and IL-6 release, MTT viability, neutrophil and macrophage migration, NF-κB and MAPK phosphorylation, and PPARγ mRNA and protein expression. | The study used compounds of reported purity, concentration-response testing, an active comparator, multiple inflammatory readouts and a mechanistic inhibitor. Nevertheless, the macrophage and zebrafish injury models are not orthopedic disease models and provide only general anti-inflammatory plausibility. The micromolar concentrations and polyphenolic stilbene structure warrant consideration of nonspecific activity and assay interference. Cell-free Griess, optical-interference and aggregation controls were not reported, although ELISA and immunoblotting provide some orthogonal support. Partial reversal by GW9662 supports PPARγ involvement but does not demonstrate direct target engagement. No mammalian pharmacokinetic or disease-efficacy data were provided. Overall concern: some concerns, with substantial indirectness for orthopedic claims. |
| **[13]** | Disease-relevant animal; composite scaffold | Composite scaffolds containing total flavonoids of pigeonpea leaf and α-calcium sulfate hemihydrate (CaS). CaS preparation was described in detail, but the pigeonpea leaf total-flavonoid fraction was identified only as being supplied by Guangzhou University of Chinese Medicine. Its botanical authentication, extraction procedure, batch, chemical profile and total-flavonoid content were not reported. | Fifty 12-week-old male SPF Sprague-Dawley rats were randomized by a random-number table into five groups (n=10): empty defect, CaS alone and three composite-scaffold groups. Bilateral 5-mm full-thickness calvarial defects were created. | A single local implantation of composite scaffolds containing pigeonpea leaf total flavonoids and CaS at mass ratios of 20:1000, 40:1000 or 80:1000 mg was performed. Assessments were conducted at 4, 8 and 12 weeks. The actual amount implanted in each defect was not stated. The 40:1000 ratio was the lowest loading reported to outperform CaS alone at the later time points. | Empty defects and CaS-only scaffolds were included. No total-flavonoid-only group, autograft control or established osteoinductive positive comparator was used. | HE and Masson staining, micro-CT reconstruction, bone mineral density and BV/TV at 4, 8 and 12 weeks. | Random allocation, a CaS component control, multiple time points and complementary histological and micro-CT assessments strengthen the study. However, the observed effect applies to the complete composite scaffold rather than to pigeonpea leaf total flavonoids in isolation. Only three rats per group were examined at each time point, and the statistical treatment of two defects from the same animal was unclear. Nine of the ten allocated animals per group were accounted for across the three time points, leaving one animal per group unexplained. Blinding, power calculation and ethical approval were not reported. Release kinetics, scaffold degradation, biomechanical properties, vascularization and systemic safety were not evaluated. A non-load-bearing calvarial defect does not reproduce long-bone nonunion or segmental bone loss, and the findings cannot be extrapolated to oral administration. Overall concern: some concerns. |

**color key (reference column):** green, human studies; blue, ONFH-related preclinical/indirect studies; yellow, osteoporosis; orange, osteoarthritis; purple, bone-defect repair.
